# Short antisense-locked nucleic acids (all-LNAs) correct alternative splicing abnormalities in myotonic dystrophy

**DOI:** 10.1093/nar/gkv163

**Published:** 2015-03-09

**Authors:** Agnieszka Wojtkowiak-Szlachcic, Katarzyna Taylor, Ewa Stepniak-Konieczna, Lukasz J. Sznajder, Agnieszka Mykowska, Joanna Sroka, Charles A. Thornton, Krzysztof Sobczak

**Affiliations:** 1 Department of Gene Expression, Institute of Molecular Biology and Biotechnology, Adam Mickiewicz University, Umultowska 89, 61-614 Poznan, Poland; 2 Department of Neurology, Box 645, University of Rochester Medical Center, 601 Elmwood Ave, Rochester, NY 14642, USA

## Abstract

Myotonic dystrophy type 1 (DM1) is an autosomal dominant multisystemic disorder caused by expansion of CTG triplet repeats in 3′-untranslated region of *DMPK* gene. The pathomechanism of DM1 is driven by accumulation of toxic transcripts containing expanded CUG repeats (CUG^exp^) in nuclear foci which sequester several factors regulating RNA metabolism, such as Muscleblind*-*like proteins (MBNLs). In this work, we utilized very short chemically modified antisense oligonucleotides composed exclusively of locked nucleic acids (all-LNAs) complementary to CUG repeats, as potential therapeutic agents against DM1. Our *in vitro* data demonstrated that very short, 8- or 10-unit all-LNAs effectively bound the CUG repeat RNA and prevented the formation of CUG^exp^/MBNL complexes. In proliferating DM1 cells as well as in skeletal muscles of DM1 mouse model the all-LNAs induced the reduction of the number and size of CUG^exp^ foci and corrected MBNL-sensitive alternative splicing defects with high efficacy and specificity. The all-LNAs had low impact on the cellular level of CUG^exp^-containing transcripts and did not affect the expression of other transcripts with short CUG repeats. Our data strongly indicate that short all-LNAs complementary to CUG repeats are a promising therapeutic tool against DM1.

## INTRODUCTION

Myotonic dystrophy type 1 (DM1) is the most common muscular dystrophy in adults, affecting 1 in 6–10 000 live birth. Clinical features of DM1 mainly present myotonia, weakness and atrophy of skeletal muscles (1). This RNA dominant disorder is caused by expansion of CTG repeats (from ∼50 to ∼3000 repeats) in the 3′ untranslated region (3′ UTR) of the *dystrophia myotonica protein kinase* (*DMPK*) gene (2). The DM1 pathogenesis is based on the dominant gain-of-function of the *DMPK* transcript containing expanded number of CUG repeats that accumulate in cell nuclei as discreet foci (3). Expanded CUG repeats (CUG^exp^) interact with RNA-binding proteins such as Muscleblind-like (MBNL) protein family, causing their nuclear sequestration and subsequently resulting in reduced protein activity in several cellular processes (4,5). Another pathogenic effect of *DMPK* mutation in DM1 is the induction of post-transcriptional upregulation of another RNA binding protein, CUGBP1 (CELF1). According to several reports, an unknown mechanism may lead to CUGBP1 phosphorylation resulting in its increased cellular stability (6). MBNLs and CUGBP1 are antagonistic regulators of alternative splicing, and both control developmentally regulated pre-mRNA maturation of many genes. Their functional imbalance leads to embryonic patterns of alternative splicing in adult DM1 tissues (7).

To date, two main experimental therapeutic strategies of targeting expanded repeat RNA in DM1 were described: (i) antisense oligomer-induced degradation of toxic CUG^exp^-containing RNA (8–16) and (ii) inhibition of pathogenic interaction of CUG^exp^ RNA with nuclear proteins (such as MBNLs) without causing significant degradation of targeted transcript, by either antisense oligomers (ASOs) (17) or small compounds that bind to CUG repeat hairpin (18). In cellular or animal models of DM, the efficient degradation of CUG^exp^ transcripts could be induced by either RNA interference (RNAi) tools (8,9), ribozymes (10), endogenously expressed long antisense CAG repeat RNAs (11) or short, chemically modified ASOs composed of CAG-repeat sequence which activate nuclear RNase H (12,14) or induce CUG^exp^ degradation according to an unknown mechanism (15,16). For an *in vivo* RNA cleavage strategy, the following oligomer types were recently used: 21-mer of phosphorothioate 2′-*O*-methyl RNA (PS-2′*O*-Me) (15), locked nucleic acid (LNA)/DNA gapmers (14), 2′-*O*-methoxyethyl (MOE)/DNA gapmers (16), siRNA duplexes (9) and nuclear expression of exogenous transcript containing CAG repeat tract fused with human U7-snRNAs (11). For an *in vivo* blocking of CUG^exp^/protein interaction, only 25-mer of CAG-25 morpholino was described so far as an efficient tool (17).

In the current study we aimed to check the potency of short synthetic oligomers composed exclusively of LNA subunits, to inhibit the toxic interaction of CUG^exp^ RNA with nuclear proteins *in vitro* and *in vivo*. The LNA is a ribonucleotide homolog with a characteristic 2′-O,4′-C-methylene bridge (19–23). LNA-containing oligomers are strict RNA mimics characterized by several attractive properties, such as high biological stability, low toxicity *in vivo*, potent biological activity and high melting temperature (*T*_m_) when bound to either DNA or RNA (19,21,23). A single LNA subunit substitution into DNA or RNA oligomer typically increases *T*_m_ by 2–8°C (19,20). Due to these attributes, nucleic acids containing additional LNA subunits have been used as ‘mixmers’ (several LNA subunits in random positions of oligomer) or ‘gapmers’ (several LNA subunits on both sides of oligomer) in many applications such as RNase H-induced gene silencing (if the central part of gapmer contains DNA nucleotides) (19,20,22,23), RNAi (23–25) or steric blocking of micro-RNA activity (21,23,26,27). In contrast, all-LNA oligomers are able to stably bind target DNA or RNA molecules without causing their endonucleolytic cleavage by RNase H (19). Previously, oligomers composed exclusively of LNA subunits (all-LNA) with only eight LNA residues were successfully applied to bind the seed regions of micro-RNAs and block their activity *in vitro* and *in vivo* (28).

Here we report the use of short antisense all-LNA oligomers to bind efficiently to long CUG^exp^ region of mutant *DMPK* transcripts in DM1 patients-derived cells and in skeletal muscle of mouse model of DM1. In cells or muscle tissue treated with all-LNAs, we observed significant reduction of nuclear CUG^exp^ foci formation, prevention of MBNLs sequestration and specific correction of alternative splicing abnormalities for several MBNL-sensitive exons, without accelerating decay of transcripts with long or short CUG repeats.

## MATERIALS AND METHODS

### Cell culture and transfection of oligomers

Fibroblasts derived from DM1 patients (cell lines GM03978, GM04033 and GM03989 expressing *DMPK* transcript with ∼500, ∼1000 and ∼2000 CUG repeats, respectively) and control fibroblasts derived from non-DM1 patients (cell line GM07492) were purchased from the Coriell Cell Repositories. Cells were grown in Eagle's minimal essential medium (EMEM) (Lonza) supplemented with 10% fetal bovine serum (FBS) (Sigma), 1% antibiotic antimycotic (Sigma) and 1% non-essential amino acids solution (Sigma), in 5% CO_2_ at 37°C. HeLa and HEK293 cells were grown in Dulbecco's modified Eagle's medium medium (DMEM) supplemented with 10% FBS (Sigma), 1% antibiotic antimycotic (Sigma) and, in 5% CO_2_ at 37°C. Control (non-DM1) myoblast line (9936) and DM1 patient-derived myoblasts (9886; 10009) were obtained from Telethon Biobank Network and were grown in F10 HAM medium (Sigma) supplemented with 20% FBS (Sigma), 0.39-μg/ml dexamethazon (Sigma), 10-ng/ml epidermal growth factor (Sigma), 25-μg/ml insulin (Sigma), 1% antibiotic antimycotic (Sigma), in 5% CO_2_ atmosphere at 37°C. Cell transfection experiments were performed using Lipofectamine 2000 (Invitrogen) according to the manufacturer's instructions with thermally denatured oligomers (in appropriate concentration) or DT960 plasmid (1 μg). For transient overexpression of *DMPK* ex11–15 (DT960CUG) HEK293 or HeLa cells were transfected with PO-LNA-CAG-10 at 125- and 250-nM final concentration, 24 h after delivery of DT960 plasmid. The total RNA was isolated 48 h after treatment or after the time specified in figure legends.

### Alternative splicing assays and semi-quantitative gene expression assays

The total RNA was isolated from cells or temporal arteritis (TA) muscles using TRI Reagent (Sigma) according to manufacturer's protocol. First strand of cDNA was synthesized using SuperScript II/III Reverse Transcriptase (Invitrogen) with Random Primers (Promega) according to manufacturer's instruction. The quality of the reverse transcription products was assessed by polymerase chain reaction (PCR) amplification of the glyceraldehyde-3-phosphate dehydrogenase (*GAPDH*) cDNA. RT-PCR was performed in using GoTaq Flexi DNA Polymerase (Promega). All PCR experiments involved at least three independent biological replicates and three technical repeats. Primer sequences and PCR conditions are specified in Supplementary Table S1. The RT-PCR products were stained with Ultra Safe Blue (Syngene) according to manufacturer's instructions and separated in 2% agarose gel. The DNA bands were visualized on G:BOX system (Syngene) and the intensity of each band was measured using GeneTools software (Syngene). EC_50_ was determined in Graph Pad Prism using log(inhibitor) versus response model: *Y* = Bottom + (Top-Bottom)/(1+10^∧^[=((*X*-LogEC_50_))). Real-time quantitative RT-PCR analysis was performed using the 7900HT Fast Real-Time PCR System (Applied Biosystems). The Power SYBR Green PCR Master Mix (Applied Biosystems) and specific primer set (0.2 μM of each) were used for amplification (Supplementary Table S1). The experiments were carried out in triplicate technical repeats for three biological replicates. The relative quantification in gene expression was determined using the ΔΔCt method. The *GAPDH*-specific signal was used as a reference.

### Quantification of normal and mutant *DMPK* transcript level

The DM1-derived myoblast line (10009) is heterozygous for *Bpm*1 polymorphism in exon 10 of *DMPK* gene (29,30). This cell line was passaged and grown in EMEM medium to selected fibroblast cells for 2 weeks. In order to distinguish the two alleles of DMPK gene, PCR product (primer—Supplementary Table S1) was digested Bpm1 enzyme (ThermoScientific) (3 h/30°C). The samples were run on a native 10% PA/TBE gel. The gel was stained with Syber Green (Invitrogen) and visualized on Gel imaging for fluorescence applications G:BOX (Syngene). The intensity of each band was measured using GeneTools software (Syngene).

### Fluorescence *in situ* hybridization

The RNA fluorescence *in situ* hybridization (FISH) was performed with the use of DNA/LNA probe (CAG)_6_-CA, labeled at the 5′-end with Cy-3 as previously described (31). Microscopic slides were mounted using medium containing 2% propyl gallate (Sigma), 10% glycerol and 4′,6-diamidino-2-phenylindole (DAPI), and then sealed with fingernail polish. All FISH images were acquired on the Nikon A1Rsi microscope equipped with the Nikon DigitalSight DS-Fi1c camera and processed with Nikon NIS Elements AR software. The quantification of RNA foci was performed using fluorescence part of microscope, filter DA/FI/TR-A-NTE (Semrock; range of excitation 387/478/555–25; emission range 433/517/613–25) and 60x oil immersion objective (Plan Apo VC 60x/1.4 Oil DIC N2). Quantification was performed by visual inspection of fluorescence spots present in cell nuclei. The number of foci was counted in at least 200 nuclei and the same experiment was repeated twice. Foci volume was estimated by Imaris software using images captured by confocal part of Nicon’ microscope in the following excitation conditions: diode lasers 405 and 561 nm, dichroic mirror 405/488/561, emission filters 450/50 for DAPI and 595/50 for Cy-3. All images were acquired with sequential scanning to avoid spectral bleed-through. Eighteen to twenty five optical sections were acquired.

### Electrophoretic mobility shift assay

The (CUG)_100_ transcript was transcribed and 5′-labeled as previously described (17). Electrophoretic mobility shift assay (EMSA) was carried out by incubating 5′ radiolabeled (CUG)_100_ (1 nM) with PO-LNA-CAG-8, PO-LNA-CAG-10, PO-LNA-CAG-12, PS-LNA-CAG-8, PS-LNA-CAG-10 or 2′*O*Me-CAG-21 of indicated concentrations (ranging from 0 to 1.25 μM). Reactions were performed in a volume of 10 μl, in 1x filter binding buffer (FBB) (50-mM NaCl, 50-mM KCl, 50-mM TRIS-HCl, 1-mM MgCl_2_, 0.05% Tween 20, pH 8.0) and incubated at 37°C for 30 min. The samples were run on a native 8% polyacrylamide (PA) gel in 0.5 x TBE at 100 V for 1 h. The gel was subsequently dried and the signal was detected O/N, visualized on IP through FLA-1500 (FujiFilm) and quantified using Multi Gauge software (FujiFilm). *K*_d_ measurement of the CUG^exp^/LNA–CAG interaction was based on the percentage of free CUG^exp^ using one phase decay curve in Graph Pad software.

### Quantification of RNA/protein interaction *in vitro*

The MBNL1 isoform carrying sequence encoded by exons 1–4 of *MBNL1* gene (NP_066368) containing glutathione-S-transferase, GST-tag, at N-terminus and His_6_-tag at C-terminus was prepared as previously described (32). Filter binding assay (FBA) was performed in a 30-μl volume. To assess the MBNL1 affinity toward distinct labeled molecules, (CUG)_100_ (0.1 nM), PO-LNA-CAG-8, PO-LNA-CAG-10 (0.5 nM) and PO-2′*O*Me-CAG-21 (0.2 nM) were incubated with the indicated concentration of the protein (ranging from 0 to 500 nM) in 1 x FBBa (FBB with 250-mM NaCl and 15-mM KCl) at 37°C for 30 min. To analyze the inhibition of MBNL1binding to (CUG)_100_ by PO-LNA-CAG and PS-LNA-CAG, 0.1 nM of labeled (CUG)_100_ was first incubated with the indicated concentrations of oligomers (ranging from 0 to 10 μM) at 37°C for 30 min, and then 15 nM of MBNL1 was added to each sample and incubated as before. Twenty-five microliter of samples was loaded onto filter binding apparatus with nitrocellulose (Protran BA 85, Whatman^®^) and nylon (Hybond™ N+, Amersham) membranes pre-wetted in 1x FBBa. The signal from membranes was visualized on IP through FLA-1500 and quantified using Multi Gauge software (FujiFilm). *K*_i_ of the prevention of (CUG)_100_/MBNL1 complexes by LNA-CAG was measured by Graph Pad using two-phase decay curve.

### Experimental mice and PO-LNA-CAG-10 treatment

Homozygous *HSA*^LR^ transgenic mice (human skeletal actin long repeat; line 20b) were previously described (33). Wild-type mice on FVB background served as controls. All animal experiments were approved by the University of Rochester Institutional Animal Care and Use Committee. Injection and electroporation was performed under general anesthesia as described previously (34). Tibialis anterior (TA) muscles of adult (2–5-month-old) mice were pretreated by intramuscular injection of bovine hyaluronidase (15 μl, 0.4 U/μl) (Sigma-Aldrich, St. Louis, MO, USA) and after 2 h, injected with 10 μg of PO-LNA-CAG-10 in 20 μl of saline, or with saline alone. Immediately following muscle injection, electroporation was performed using electrodes placed parallel to the long axis of the muscle. Electroporation parameters were 100 V/cm, 10 pulses at 1 Hz and 20 ms duration per pulse. Northern blot analysis of CUG^exp^ transcripts and RT-PCR assays monitoring alternative splicing of *Atp2a1, Titin* and *Nfix* were performed after 2 weeks from PO-LNA-CAG-10 injection as described previously (17). Electromyography (analysis of myotonia) was performed under general anesthesia as described (17). Myotonia was graded as follows: 0 indicates no myotonia; 1, occasional myotonic discharge in less than 50% of electrode insertions; 2, myotonic discharge in greater than 50% of insertions; 3, myotonic discharge with nearly every insertion.

### Statistical analysis

Group data are expressed as mean ± standard deviation. Statistical significance of RT-PCR results was determined by a two-tailed Student's *t*-test using Microsoft Excel (*P < 0.05; **P < 0.01 and ***P < 0.001).

## RESULTS

One of the pathogenic effect of a CTG repeat expansion is an undesirable interaction of toxic CUG^exp^ RNA with several nuclear proteins (4). In this study, we tested whether very short antisense oligomers composed exclusively of LNA subunits can efficiently bind to long CUG^exp^ hairpin structure *in vitro* and *in vivo*, in order to inhibit pathogenic RNA/protein interaction without causing activation of degradation pathways for long or short CUG repeats-containing transcripts. The stable occupation of CUG^exp^ by LNA is meant to block the excessive binding of proteins such as MBNLs, hence restoring their normal availability in cellular environment and rescuing cellular processes in which they are involved, such as the alternative splicing regulation.

### *In vitro* screening of antisense all-LNA oligomers complementary to CUG repeats

To determine the minimal length of oligomer composed exclusively of LNA residues sufficient to bind a CUG^exp^ hairpin structure *in vitro* and to correct some of the DM1-specific phenotypes in cellular models, we designed a series of all-LNA oligomers having phosphodiester linkages and differing in the length of the sequence complementary to CUG repeats (Table 1). These included: a 6-mer (PO-LNA-CAG-6), an 8-mer (PO-LNA-CAG-8), a 10-mer (PO-LNA-CAG-10) and a 12-mer (PO-LNA-CAG-12). The predicted melting temperatures (*T*_m_) of designed oligomers differ significantly and amount to 19, 35, 49 and 56^o^C, respectively (35). We expected that the thermodynamic parameters of PO-LNA-CAG-8, PO-LNA-CAG-10 and PO-LNA-CAG-12 could be sufficient for specific interaction with CUG repeats. The oligomers longer than 12 LNA subunits, with significantly higher *T*_m_, could bind non-specifically to RNA or DNA targets and could form stable intermolecular duplexes and hairpin structures, which could reduce their activity.

**Table 1.** The list of antisense oligomers

Oligomer name	Sequence 5′-3′^a^	RNA *T*_m_ [°C]^b^
PO-LNA-CAG-6	CAGCAG	19
PO-LNA-CAG-8	CAGCAGCA	35
PO-LNA-CAG-10	CAGCAGCAGC	49
PO-LNA-CAG-12	CAGCAGCAGCAG	56
PS-LNA-CAG-8	**CAGCAGCA**	-
PS-LNA-CAG-10	**CAGCAGCAGC**	-
PO-LNA-CTR1	GTGGAAGCGG	57
PO-LNA-CTR2	GGCACAAGCG	52
PO-LNA-CTR3	ACGCACAAGG	65
PO-2′*O*Me-CAG-21	^m^C^m^A^m^G^m^C^m^A^m^G^m^C^m^A^m^G^m^C^m^A^m^G^m^C^m^A^m^G^m^C^m^A^m^G^m^C^m^A^m^G	75

^a^‘Upper case’ denotes LNA modification; ‘underline and bold’, PS modification; ‘^m^C; ^m^A; ^m^G’, 2′*O*Me modification.

^b^*T*_m_, prediction of melting temperature for oligomer/RNA duplex is based on www.exiqon.com/ls/Pages/ExiqonTMPredictionTool.aspx.

**Figure 1.**
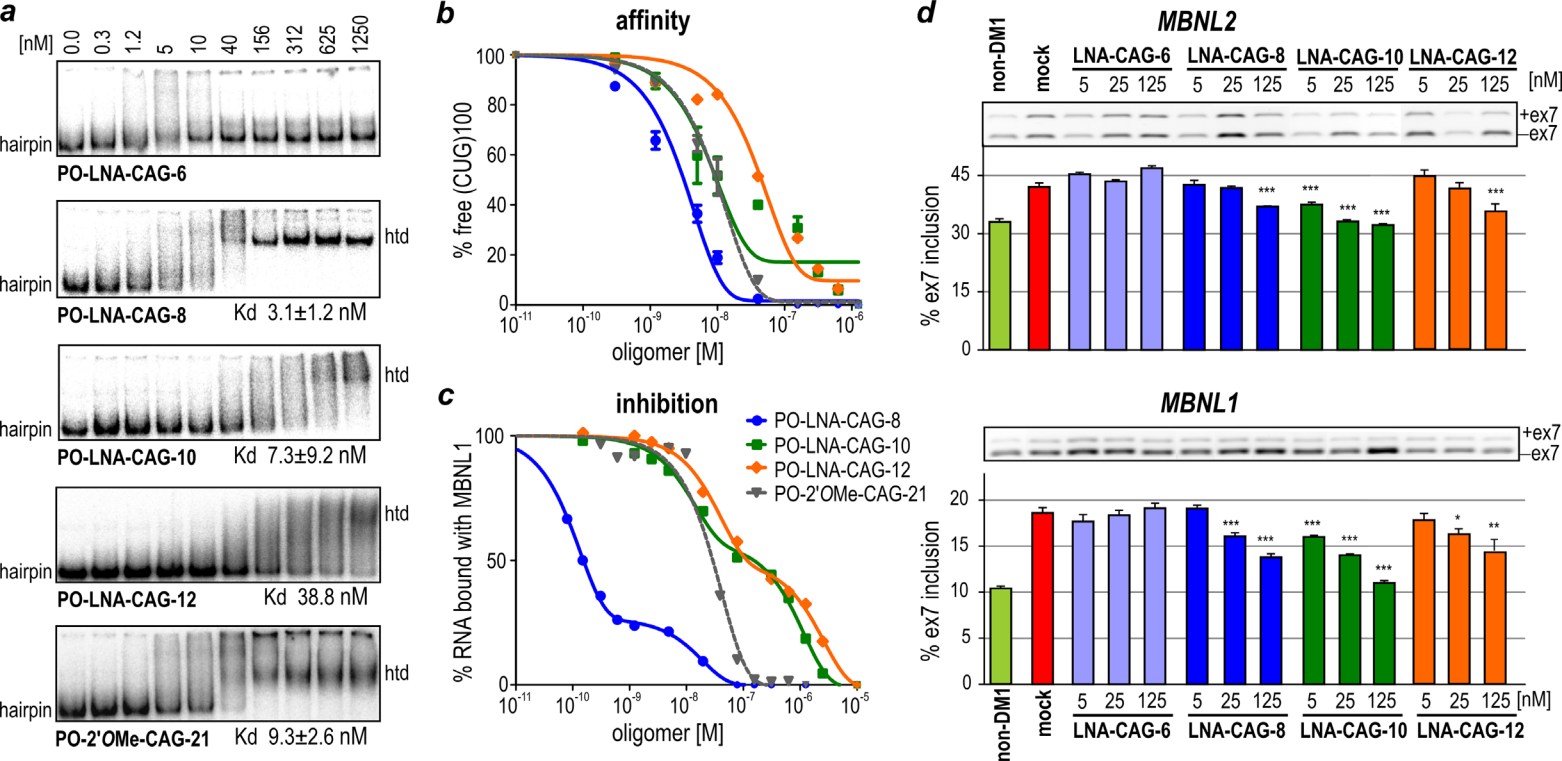
8, 10 or 12-unit antisense LNA oligomers efficiently bind stable CUG^exp^ hairpin and correct MBNL-sensitive alternative splicing. (**a**) EMSA on a native polyacrylamide gel showing the interaction between distinct PO-LNA-CAG oligomers used at indicated concentrations and a 5′-end radiolabeled (CUG)_100_ used at a concentration of 0.1 nM. Migration of RNA hairpin (hairpin) and heteroduplexes of RNA with several oligomers (htd) is indicated. PO-LNA-CAG-6 is unable to form full complexes with (CUG)_100_; however, PO-LNA-CAG-8 forms such complexes at much lower concentration than both PO-LNA-CAG-10 and PO-LNA-CAG-12. The *K*_d_ value for each interaction, based on at least three independent assays, is specified bellow each electrophoregram. 2′*O*Me-CAG-21 oligomer was used as a control. It shows that different modifications and length of molecules slightly change the strength of their interaction with long CUG repeat RNA. (**b**) The quantification of EMSA results based on the decline of free (CUG)_100_ in favor of forming CUG_100_/LNA-CAG complexes. (**c**) Prevention of the (CUG)_100_/MBNL1 interaction in the presence of tested PO-LNA-CAGs. The curves show inhibition of MBNL1 binding by all tested oligomers. (**d**) RT-PCR results of alternative splicing changes of *MBNL2* and *MBNL1* transcript in non-DM1 fibroblasts (first lane) and (CUG)1000 fibroblasts treated with transfection reagent only (mock) or one of the four oligomers (PO-LNA-CAG-6, PO-LNA-CAG-8, PO-LNA-CAG-10 and PO-LNA-CAG-12) used at three different concentrations (5, 25, 125 nM). Upper bands represent exon inclusion isoforms (+ex7) while lower bands represent exon exclusion (−ex7). Lower level of alternative exon inclusion after treatment with highest concentrations of PO-LNA-CAG-8 and PO-LNA-CAG-12, and with all tested PO-LNA-CAG-10 concentrations, indicates alternative splicing correction. Statistical significance was determined using two-tailed Student's *t*-test by comparison of average results from three independent experiments to results obtained for mock samples (*P < 0.05, **P < 0.01 and ***P < 0.001).

In the initial *in vitro* assays we tested whether any of the designed all-LNAs are able to bind the thermodynamically stable RNA hairpin structure formed by long CUG repeats and inhibit its interaction with recombinant MBNL1. Using EMSA, we monitored the affinity of tested oligomers to an *in vitro* transcript composed of ∼100 CUG repeats, referred to as (CUG)_100_ (Figure 1a). The shortest oligomer did not invade the (CUG)_100_ hairpin but unexpectedly, PO-LNA-CAG-8 showed slightly higher affinity toward the hairpin than longer PO-LNA-CAG-10, and much higher than PO-LNA-CAG-12. The dissociation constant values (*K*_d_) for 8-mer and 10-mer oligomers were very low and accounted to ∼4 and ∼7 nM, respectively (Figure 1a and b). PO-LNA-CAG-12 appeared to have five to ten times higher *K*_d_ (40 nM). As control, we used long 21-mer CAG repeat oligomer, with 2′-*O*-methyl modification in each position (PO-2′*O*Me-CAG-21) with predicted *T*_m_ of 75°C. The *K*_d_ value for control oligomer was within the same range as for all-LNAs (5–10 nM).

Next, we asked whether tested oligomers have the potential to inhibit CUG repeat RNA/MBNL1 interaction *in vitro*. For this purpose we first used FBA and measured the affinity of MBNL1 to the transcript containing 35 CUG repeats (CUG)_35_, derived from normal *DMPK* allele, and a three times longer mutant transcript, (CUG)_100_. MBNL1 bound both types of transcripts with similar affinity (Supplementary Figure S1a and b). Then, using the same method, we tested the interaction between radiolabeled (CUG)_100_ and MBNL1 in the absence or presence of each oligomer. PO-LNA-CAG-8 showed the lowest inhibition constant (*K*_i_) which was about 100-fold lower than *K*_i_ for a 10-mer and 300-fold lower than *K*_i_ for a 12-mer. The inhibitory potential of the control 2′*O*Me-CAG-21 oligomers resembled the one observed for PO-LNA-CAG-12 (Figure 1c).

One of the possible explanations for such differences might be the competition of CUG hairpin with CAG repeat oligomers, since previous studies reported that short CUG or CAG repeat containing RNA oligomers can bind MBNL1 *in vitro* with relatively low *K*_d_ (36). Therefore, we tested the ability of PO-LNA-CAGs and control oligomers to bind recombinant MBNL1 using FBA. We did not, however, observe any interactions between MBNL1 and tested oligomers in the 0–500-nM protein range (Supplementary Figure S1c and d).

### Screening of antisense all-LNA oligomers in DM1 cells

To assess the potency of all-LNA oligomers in a physiologically relevant model of DM1, we employed three DM1 patients-derived fibroblast cell lines expressing mutant *DMPK* transcripts with ∼500, ∼1000 and ∼2000 CUG repeats, referred to as (CUG)500, (CUG)1000 and (CUG)2000, respectively. These cells display several DM1-specific molecular phenotypes, such as the formation of CUG^exp^ nuclear foci, sequestration of MBNL1 and aberrant alternative splicing of several MBNL-sensitive exons. In an initial attempt to find alternative exons that could serve as useful markers for splicing correction analyses in DM1 cells, we examined roughly 20 alternatively spliced transcripts known to be sensitive to MBNL level, and ∼20 transcripts regulated by other splicing factors either affected in DM1 (CUGBP1) or not affected in DM1 (PTBP1, PTBP2, NOVA1 and FOX2—used as the negative controls). The reverse transcription PCR (RT-PCR) assays showed that most of the tested alternative splicing events were not informative in our cellular models due to either low expression level or presence of only one alternative splicing isoform (100% of either exon inclusion or exon exclusion isoform) (Supplementary Figure S2a). For further studies we chose only six MBNL-sensitive splicing events, namely *MBNL1* exon 7 (7), *NFIX* exon 7 (37), *INSR* exon 11 (38), *MBNL2* exon 7, *PHKA1* exon 19 and *NCOR2* exon 45a (39) (Supplementary Figure S2b). Previously, it was reported that all of these were misregulated in skeletal muscles of DM1 patients and *Mbnl1* knockout mice (39), but also were sensitive to silencing of *MBNL1* in DM1 and non-DM1 fibroblasts (40). Simultaneously, we selected five MBNL-independent splicing events as controls. They include either exons affected in DM, namely *SOS1* exon 26 and *KIF13A* exon 21 (39) or unaffected in DM, *PPP3CB* exon 13 (PTBP2-sensitive) (41), *APLP2* exon 14 (NOVA1-sensitive) (42), *ECT2* exon 4 (FOX2-sensitive) (43) (Supplementary Figure S2c). In our cellular models we did not, however, observe any differences in splicing pattern of all these MBNL-independent exons between DM1 and non-DM1 cells (Supplementary Figure S2c). Moreover, splicing events misregulated in DM muscles, *KIF13A* exon 21 and *SOS1* exon 26, exist only in muscle-specific isoform in all of the analyzed cell lines.

We then verified the cellular localization of fluorescently labeled LNA-containing oligomers after single transfection with the use of Lipofectamine 2000. These oligomers localized predominantly in cell nuclei, especially after longer time from transfection (Supplementary Figure S3). These data suggest that short all-LNAs could potentially be able to interact with CUG^exp^ transcript located exclusively in the nucleus and may have limited effect on other cytoplasmic transcripts containing CUG repeats.

Next, we transfected two DM1 cell lines, (CUG)1000 and (CUG)2000, with three different concentrations of each PO-LNA-CAG oligomer (5, 25 and 125 nM). After 48 h from single transfection, we analyzed two MBNL1-regulated splicing events. For both assayed transcripts, *MBNL1* and *MBNL2*, we observed a shift from DM1-specific splicing isoform (alternative exon inclusion) toward normal, non-DM1 isoform (alternative exon exclusion) in cells transfected with 125 nM of PO-LNA-CAG-8, 125 nM of PO-LNA-CAG-12 and all tested concentrations of PO-LNA-CAG-10 (Figure 1d).

As a control we used PO-2′*O*Me-CAG-21 oligomer which showed high affinity toward CUG repeat RNA hairpin in our *in vitro* tests. Similar oligomer, but with additional phosphorothioate modification (PS-2′*O*Me), was previously shown in DM1 models as an effective tool inducing degradation of expanded CUG repeat RNA (15). We did not observe any influence of PO-2′*O*Me-CAG-21 on splicing changes of MBNL-sensitive exons in DM1 cells (Supplementary Figure S4), and we hypothesize that this could be caused by lower stability of phosphodiester containing oligomer compared to its PS-modified version. As the PO-LNA-CAG-10 showed the highest potency, we have decided to use it for all further analyses.

### PO-LNA-CAG-10 specifically corrects MBNL-sensitive alternative splicing abnormalities

**Figure 2.**
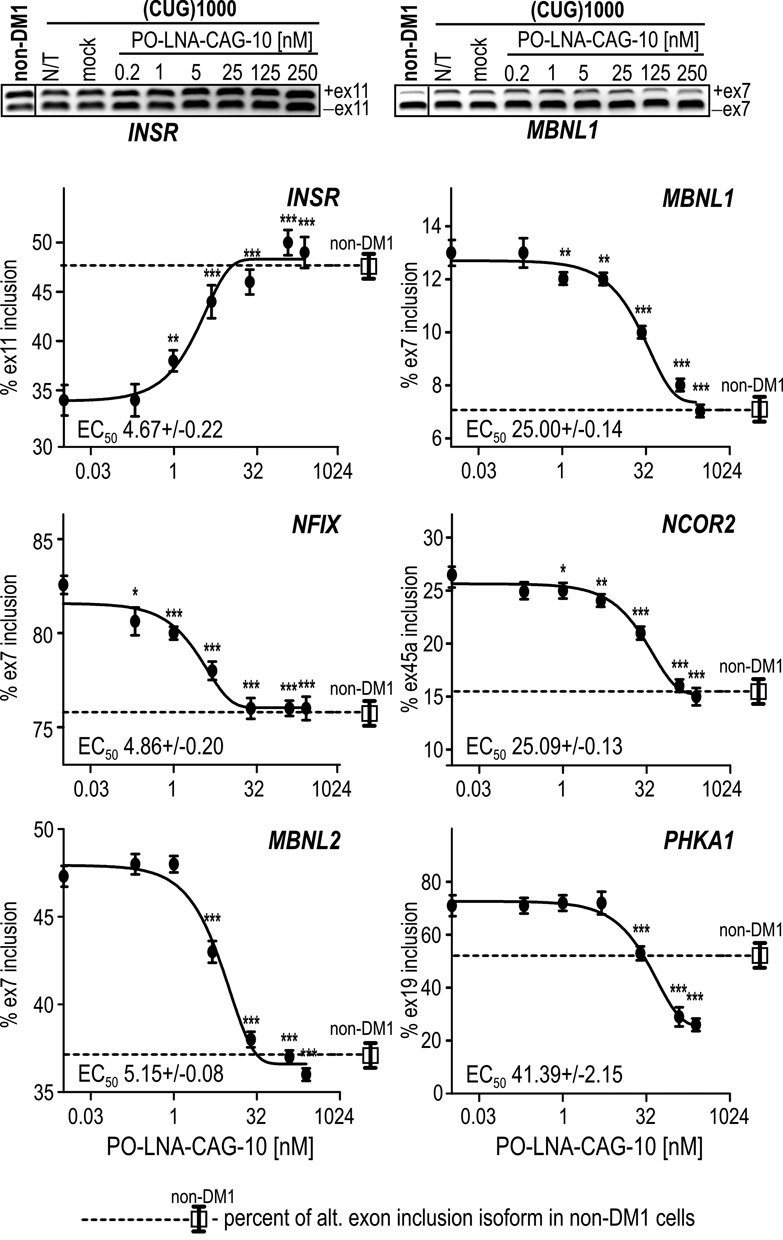
PO-LNA-CAG-10 corrects alternative splicing of MBNL-sensitive exons at low nanomolar concentrations. Results of RT-PCR analyses for alternatively spliced exons of *INSR, NFIX, MBNL2, MBNL1, NCOR2*, and *PHKA1* transcripts in (CUG)1000 cells treated with 0.2–250-nM PO-LNA-CAG-10. The splicing pattern of all tested alternative exons in untreated non-DM1 cells is also shown. The EC_50_ values were calculated for each transcript. Other details are the same as described in Figure 1d. Statistical significance was determined using two-tailed Student's t-test by comparison of average results from three independent experiments to results obtained for mock samples (*P < 0.05, **P < 0.01 and ***P < 0.001).

To be able to characterize the potency of PO-LNA-CAG-10 more precisely, we tested the broad range of oligomer concentrations (0.2–250 nM) in DM1 fibroblasts. We monitored alternative splicing correction of six studied MBNL-sensitive splicing events 48 h after transfection. All tested exons were sensitive to low nanomolar concentrations of PO-LNA-CAG-10. LogEC_50_ values ranged from ∼5 nM (for alternative exons of *MBNL2, NFIX* and *INSR*) to ∼40 nM (for exon 19 of *PHKA1*). At 125 nM of PO-LNA-CAG-10, most tested transcripts showed splicing pattern characteristic for non-DM1 cells (Figure 2), in contrast to PO-LNA-CAG-8 which gave similar effects in considerably higher concentrations (Supplementary Figure S5).

In order to determine specificity of PO-LNA-CAG-10, we designed three control oligomers containing 10 LNA residues: scrambled sequence with the same but mixed nucleotide composition (PO-LNA-CTR3) and two other oligomers (PO-LNA-CTR1 and PO-LNA-CTR2) with random sequences (Table 1). We observed that all control LNA oligomers transfected at 125 nM either did not have any effect on splicing of tested MBNL-sensitive transcripts, or their presence in cells shifted the splicing pattern toward the DM1-like (Supplementary Figure S6).

Next, we asked whether LNA oligomers could impact alternative splicing more globally. For this purpose, we analyzed five MBNL-independent alternative splicing events in the same DM1 cells. We did not observe any significant alternative splicing changes after treatment with PO-LNA-CAG-10 (Supplementary Figure S7). These results suggest that PO-LNA-CAG-10 very specifically corrects the alternative splicing of MBNL-sensitive exons in DM1 cells, most probably due to its high binding specificity toward CUG^exp^ hairpin in mutant *DMPK* transcript and inhibition of toxic CUG^exp^/MBNLs interaction. Conversely, splicing pattern of alternative exons of three genes, *PPP3CB, SOS1* and *KIF13A*, changed significantly after treatment with some of the control LNAs.

**Figure 3.**
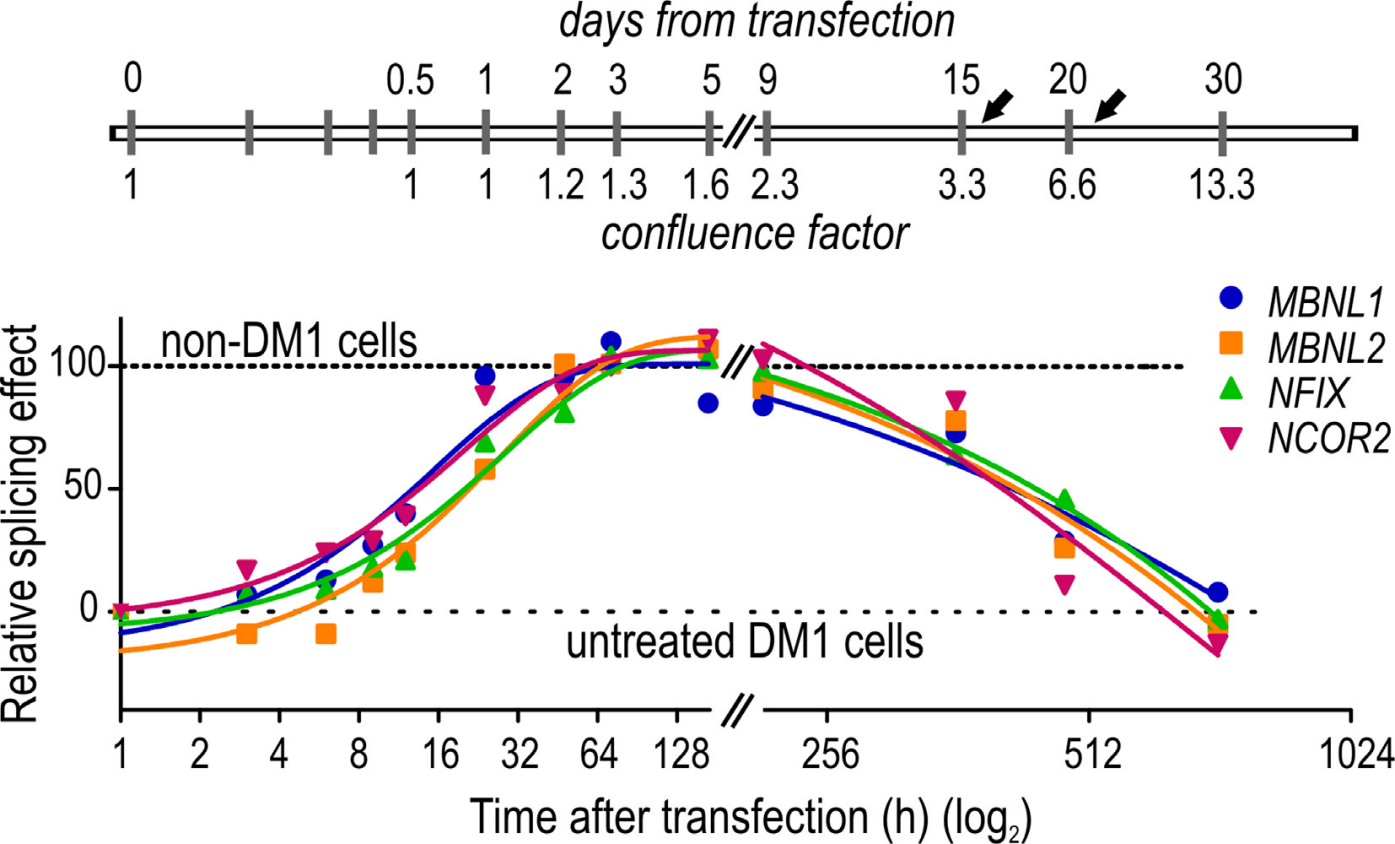
Timeline of alternative splicing correction in PO-LNA-CAG-10-treated DM1-cells. Longitudinal analysis of alternative splicing changes in *MBNL1, MBNL2, NFIX* and *NCOR2* transcripts in (CUG)1000 fibroblasts after 3, 6, 9, 12, 24 h and 2, 3, 5, 9, 15, 20 and 30 days from single transfection with 125-nM PO-LNA-CAG-10. Cells were passaged twice at days 16 and 21 (indicated by black arrows), at which the numbers of cells were four times and eight times higher, respectively, than at the time of transfection (confluence factor is indicated below timeline). The relative splicing effect shows the percentage of alternative exon inclusion at different experimental time points. Horizontal lines indicate the threshold characteristic for non-DM1 cells (value ‘100’) and not treated (CUG)1000 cells (DM1 non-treated; value ‘0’). The most significant splicing correction effect was observed between ∼24 and ∼216 h post transfection.

Finally, to determine the duration of PO-LNA-CAG-10 activity in dividing cells, we performed a time-course experiment (Figure 3). The DM1 fibroblasts at ∼30% confluence were transfected with 125 nM of PO-LNA-CAG-10 and alternative splicing events of four MBNL-sensitive exons were analyzed from 3 h to 30 days post transfection. The maximal effect of PO-LNA-CAG-10, leading to the splicing pattern observed in non-DM1 cells, was acquired after 2 days from transfection. After 15 days the correction effect was ∼75% of maximal effect, despite cell divisions. Five days later the splicing pattern shifted to that of not treated DM1 cells (Figure 3). These results indicate that PO-LNA-CAG-10 is stable in cellular environment and active even after its dilution caused by cell proliferation.

### PO-LNA-CAG-10 significantly reduces the number of CUG^exp^-containing foci without degradation of toxic CUG^exp^ RNA

**Figure 4.**
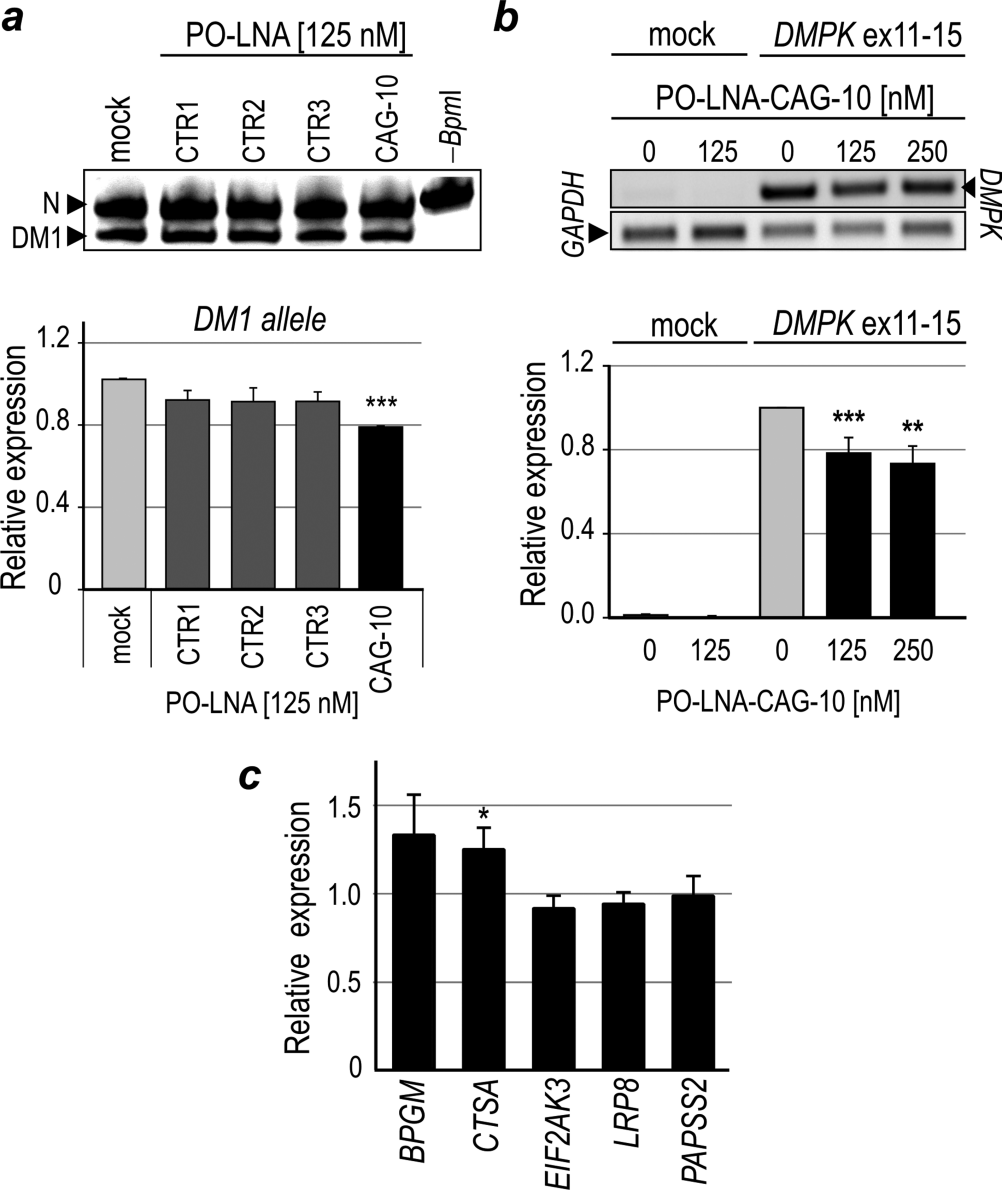
The level of CUG^exp^ RNA is slightly decreased in PO-LNA-CAG-10-treated DM1 cells. (**a**) Allele-specific semi-quantitative RT-PCR assay measuring the relative level of transcripts from two *DMPK* alleles in DM1 fibroblasts transfected with 125 nM of each of the three control PO-LNAs (CTR1, CTR2 and CTR3) or PO-LNA-CAG-10. RT-PCR products containing *Bpm*I polymorphism were either digested with *Bpm*I (first five lanes) or undigested (-*Bpm*I; last lane). DM1-specific RT-PCR product is sensitive to *Bpm*I (DM1; lower band) and normal, non-DM1 variant is resistant to *Bpm*I (N; upper band). (**b)** The relative level of the DMPK transcripts containing 960 CUG repeats in HeLa cells transiently transfected with DT960 expression or treated with transfection reagent only (mock). Cells were treated with either transfection reagents (lane 0) or PO-LNA-CAG-10 at two concentrations (125 and 250 nM). The results were normalized to *GAPDH*-specific RT-PCR product. Note that only slightly decreased levels of CUG^exp^ containing RNA were observed in (a) and (b) upon PO-LNA-CAG-10 treatment. (**c**) The relative level of transcripts containing short CUG repeat tracts (from 7 to 12 repeats) after PO-LNA-CAG-10 treatment was measured by real-time RT-PCR (normalized to *GAPDH* mRNA). The level of all transcripts in mock-treated samples was fixed as 1 and used for statistical analyses. Neither of the tested transcripts showed significant splicing change in the presence of LNA oligomer. (*P < 0.05, **P < 0.01 and ***P < 0.001).

Previously published studies have shown that various antisense oligomers targeting CUG repeats, either in cellular or animal DM1 models, induce degradation of CUG^exp^-containing transcript (8–16). To determine whether PO-LNA-CAG-10 present in nuclei of DM1 cells causes degradation of mutant *DMPK* transcript we employed DM1 cells heterozygous for BpmI restriction site polymorphism present in exon 10 of *DMPK* (29,30). Both fibroblastic and myoblastic *BpmI*-polymorphic DM1 cell lines were transfected with 125 nM of PO-LNA-CAG-10. The semi-quantitative RT-PCR-BpmI restriction fragments length polymorphism analyses showed only very mild (∼20%) reduction of mutant *DMPK* mRNA level in treated cells (Figure 4a). Moreover, there was no significant reduction of total level of *DMPK* transcript in these cells (data not shown). Additionally, we tested the effect of PO-LNA-CAG-10 on artificial transcript containing CUG^exp^ in HEK293 and HeLa cells transiently transfected with DT960 vector expressing *DMPK* exons 11–15, with 960 interrupted CTG repeats in exon 15 (44). We observed that the delivery of PO-LNA-CAG-10 into these cells resulted in only ∼20% decrease in expression of artificial *DMPK* transcript (Figure 4b). Taken together, these data demonstrate that the tested LNA oligomer causes only slight but statistically significant reduction in the level of CUG^exp^ containing transcripts.

We also analyzed the expression level of several other transcripts containing short CUG repeat tracts in (CUG)1000 cells and found that neither of the five tested transcripts, *BPGM, CTSA, EIF2AK3, LRP8* and *PAPSS2*, showed statistically significant reduction of expression level after treatment with 125 nM of PO-LNA-CAG-10. The only exception was the *CTSA* transcript having significantly higher cellular level (Figure 4c and Supplementary Figure S8). These results strongly support the specificity of PO-LNA-CAG-10 toward mutant *DMPK* transcript and demonstrate that all-PO-LNA-CAGs have no impact on the level of transcripts containing complementary sequences in distinct parts of unrelated transcripts.

**Figure 5.**
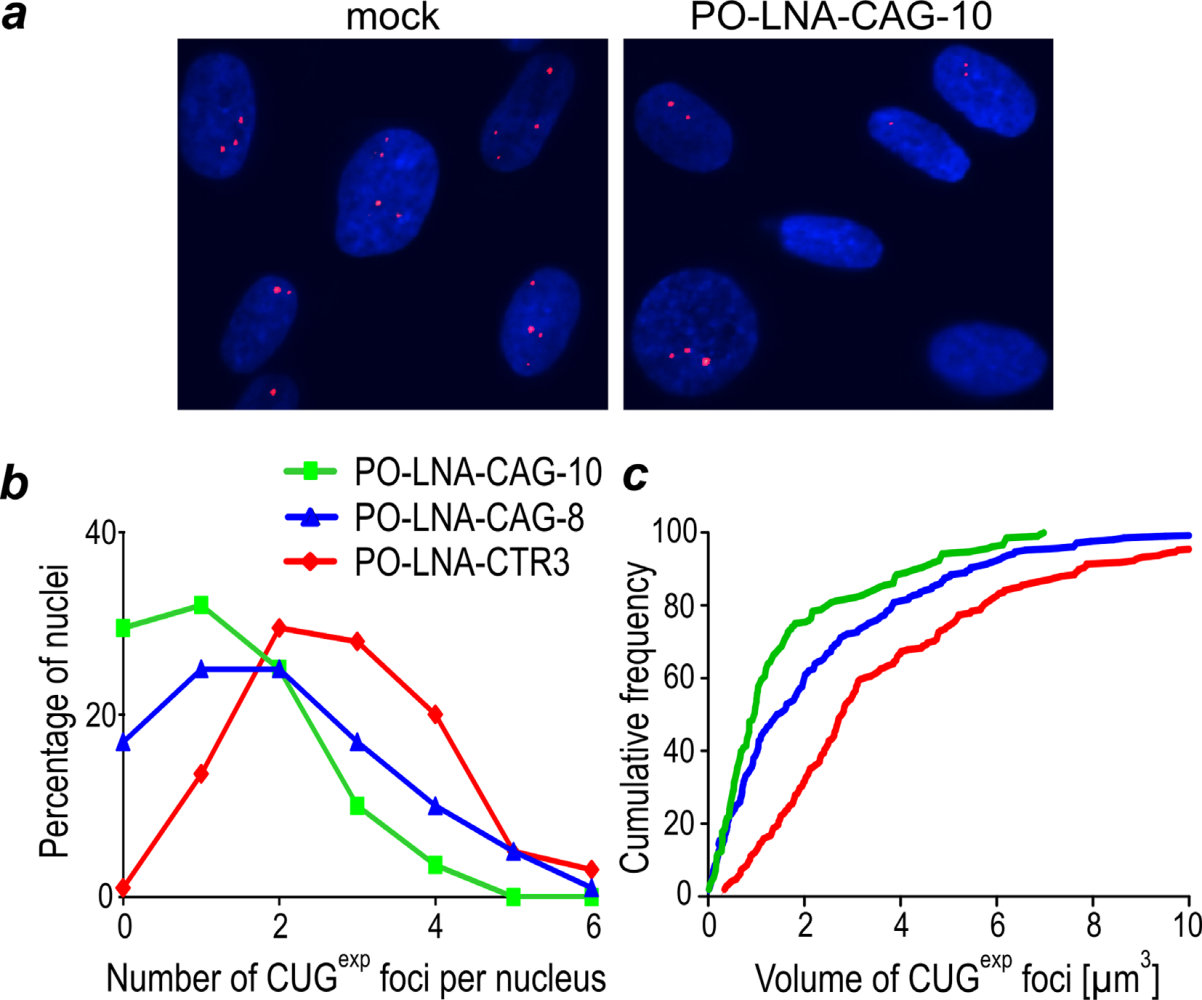
The number and size of CUG^exp^ containing nuclear foci are significantly decreased in PO-LNA-CAG-10-treated DM1 cell. (**a**) Representative FISH analyses of CUG^exp^ foci in (CUG)1000 fibroblasts treated with transfection reagent (mock) or PO-LNA-CAG-10 (125 nM). The average number of foci per nucleus (**b**) and their average volume (μm^3^) (**c**) were measured in 400 arbitrary-selected nuclei from cells transfected with 125 nM of either control PO-LNA-CTR3, PO-LNA-CAG-8 or PO-LNA-CAG-10 oligomers.

Next, we asked whether the interaction of PO-LNA-CAG-10 with expanded CUG repeats in DM1 cells could influence the number and size of nuclear CUG^exp^ foci. Three DM1 cell lines differing in CUG-repeat expansion size, (CUG)500, (CUG)1000 and (CUG)2000, respectively, were treated for 48 h with PO-LNA-CAG-10, PO-LNA-CAG-8 or control oligomers. The results of RNA fluorescent *in situ* hybridization (FISH) revealed a significant reduction in the average number of nuclear CUG^exp^ foci. In striking contrast to untreated or control LNA-treated cells which contained ∼60% of nuclei with three or more foci, all DM1 cells treated with PO-LNA-CAG-10 contained only ∼15% of such nuclei (Figure 5a and b and Supplementary Figure S9). Moreover, as much as ∼60% of PO-LNA-CAG-10-treated cells and only ∼15% of control LNA-treated cells contained no CUG^exp^ foci or only a single focus. We also observed statistically significant reduction of the size of CUG^exp^ foci in cells treated with PO-LNA-CAG-10 compared to mock-treated cells. The median volume of RNA foci amounted to 0.97 and 2.79 μm^3^, respectively (Figure 5c). Significant but slightly lower effects on CUG^exp^ foci were observed for cells treated with the same concentration of PO-LNA-CAG-8 (Figure 5b and c). Our results indicate that the interaction of PO-LNA-CAGs with mutant *DMPK* transcript does not influence cellular degradation of targeted RNAs but instead, significantly, reduces both the number and the size of toxic CUG^exp^ foci, most probably due to dispersion of the mutant *DMPK* transcripts in these cells.

### Phosphorothioated LNA oligomers do not correct alternative splicing after unassisted delivery into DM1 cells

Recently, Stein and collaborators used an intracellular delivery method called gymnosis (45) that does not require the use of any transfection reagent or conjugates, but rather relies on unassisted free uptake of ‘naked’ oligomers added directly to cell culture medium. Although gymnosis was originally demonstrated using phosphorothioated LNA (PS-LNA) gapmers (45), efficient unassisted uptake was also described for unmodified all-PS-LNAs, termed ‘tiny LNAs’ (28,46,47). These reports encouraged us to test whether gymnosis could be utilized to deliver LNA-CAGs. Supply LNA-CAGs to cells without any carriers could increase the chances of systemically delivery oligomers *in vivo*. Our experiments demonstrated that neither PO-LNA-CAG-8 nor PO-LNA-CAG-10 was able to get into cells without transfection reagents (data not shown). Therefore, we wondered whether additional chemical modification of these oligomers could improve their bioavailability. For this purpose, two fully phosphorothioated all-LNAs consisting of either 8 (PS-LNA-CAG-8) or 10 (PS-LNA-CAG-10) LNA residues first were tested *in vitro*. The EMSA and FBA showed that both oligomers have very high affinity to long CUG repeat hairpin (*K*_d_ = 0.8 nM for PS-LNA-CAG-8 and 5 nM for PS-LNA-CAG-10) and strong inhibitory effect on MBNL1 binding (*K*_i_ = 3.2 nM for PS-LNA-CAG-8 and 262 nM for PS-LNA-CAG-10), which are comparable with PO-LNA-CAG-8 and PO-LNA-CAG-10 (Supplementary Figure S10).

To analyze the potency of PS-LNA oligomers in cells, we first delivered them by lipofection. PS-LNAs were able to induce alternative splicing correction to a similar extent as unmodified PO-LNA-CAGs (Supplementary Figure S11a). Next, fully phosphorothioated LNA oligomers were delivered gymnotically into (CUG)1000 fibroblasts by incubating the cells for 72 or 216 h in a medium containing from 0.5 to as much as 10 μM of a specific oligomer. Neither experiment carried out for 72 h caused any significant alternative splicing changes of four tested MBNL-sensitive exons (data not shown). However, three times longer treatment with PS-LNA-CAG-8, at 10-μM concentration but not at 0.5-μM concentration, resulted with slight but statistically significant correction of three out of the five analyzed splicing events (exons 7, 45a and 11 of *MBNL2, NCOR2* and *INSR*, respectively) (Supplementary Figure S11b). Moreover, FISH analyses of CUG^exp^ foci in DM1-fibroblasts revealed that gymnotically delivered PS-LNAs reduced the average number of CUG^exp^ foci in DM1 cells after both, 72- and 216-h-long treatment. The reduction was minor after 72 h, however, upon extended incubation time the effect was much more prominent and similar to the one observed upon canonical transfection of 25-nM PS-LNA with lipofectamine (Supplementary Figure S11c). Taken together, our data confirm that PS-LNAs can be effectively delivered into cells by gymnosis, but to reach comparable effectiveness as with lipofectamine-delivered PS-LNAs, the concentration of the oligonucleotides must be two orders of magnitude higher.

### PO-LNA-CAG-10 corrects alternative splicing in DM1 muscle cells and in DM1 mouse model

**Figure 6.**
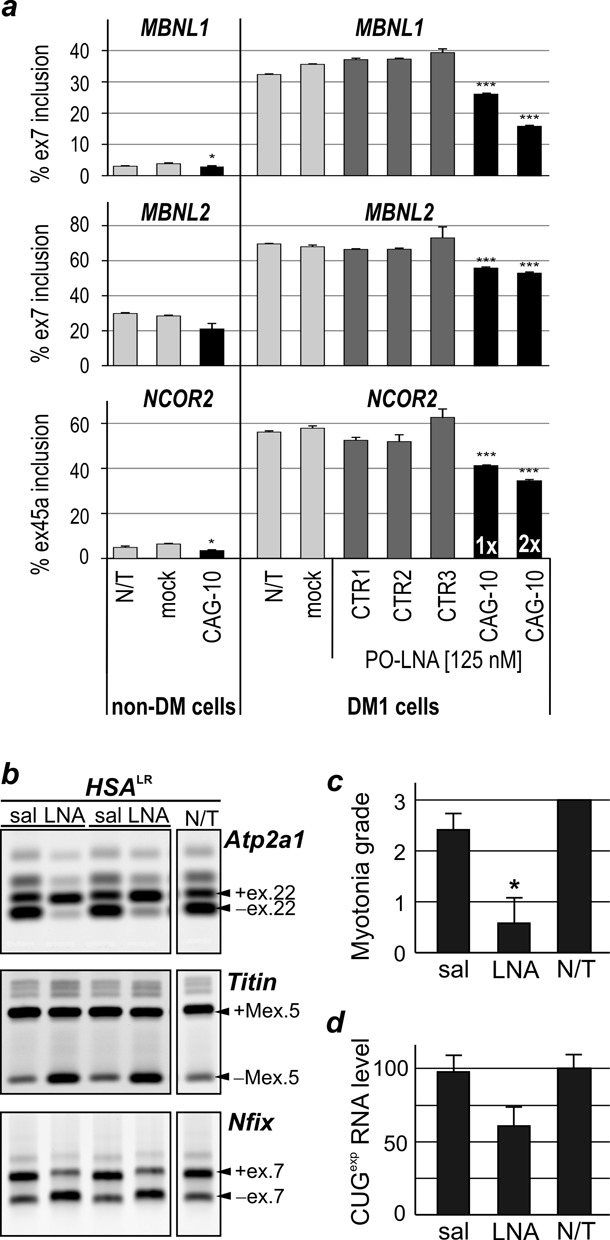
PO-LNA-CAG-10 corrects missplicing in DM1 muscle cells and in mouse model of DM1. (**a**) Alternative splicing analyses after a single (1x) or double (2x) transfection of 125-nM control PO-LNAs (CTR1, CTR2, CTR3) and PO-LNA-CAG-10 in myoblasts cells derived from non-DM1 individual (non-DM1) and DM1 patient. The non-treated cells (N/T) and cells treated with transfection reagent (mock) were used as controls. Other details are the same as described in Figure 1d. Note that for control LNAs-treated cells there are no significant differences in splicing isoform distribution. (**b**) Tibialis anterior muscles of two *HSA*^LR^ mice were injected with either saline (sal) or 5 μg of PO-LNA-CAG-10 (LNA). The splicing pattern of exons regulated by Mbnl1 of three transcripts (*Atp2a1, Titin* and *Nfix*) was analyzed 14 days post-treatment. Non-treated muscle (N/T) of an *HSA*^LR^ mouse was used as a control. (**c**) Myotonic discharges were measured for each treated and non-treated muscle and the grade of myotonia was arbitrary classified using scale from 0 to 3. The results are presented as an average from two to four animals. (**d**) The relative level of CUG^exp^ containing transcript was quantified by norther blot hybridization. (*P < 0.05, **P < 0.01 and ***P < 0.001).

To test all-LNA oligomers in more physiologically relevant DM1 models we used both the DM1 patient-derived muscle cells and a DM1 mouse model. The DM1 and non-DM1 myoblasts were transfected with 125 nM of PO-LNA-CAG-10 and three control all-LNA oligomers. After 48 h, we observed a significant correction of the alternative splicing pattern of *MBNL1* exon*7, MBNL2* exon*7* and *NCOR2* exon*45a* only in PO-LNA-CAG-10-treated DM1 cells (Figure 6a). However, the percentage of exon inclusion indicative of the DM1-like splicing perturbations, although significantly reduced in PO-LNA-CAG-10-treated DM1 cells, did not decline to the level observed in non-DM1 myoblasts. Importantly, the correction effect was stronger when two consecutive transfection procedures were applied. Conversely, no significant differences in alternative splicing pattern of tested exons were observed in cells transfected with either of the control all-LNA oligomers (Figure 6a). These data strongly support the specificity and potency of PO-LNA-CAG-10.

Finally, we tested the activity of PO-LNA-CAG-10 in *human skeletal actin-*long repeat (*HSA*^LR^) mouse model in which skeletal muscles express the human alpha actin transcript containing ∼220 CUG repeats in the 3′ UTR. This mouse model shows a number of molecular features observed in DM1 patients, including formation of nuclear CUG^exp^ foci that sequester Mbnl proteins, alternative splicing abnormalities of Mbnl-sensitive exons and myotonic discharges in skeletal muscles (33). The TA muscles of two *HSA*^LR^ mice were injected with 5 μg of the PO-LNA-CAG-10 and the TA in the opposite hind limb was injected with saline only. Following the injection, each muscle was electroporated as previously described (34) to facilitate entry of oligomer into muscle fibers. Mice were sacrificed for analyses two weeks after a single oligomer injection. The RT-PCR assays were used to monitor an effect of PO-LNA-CAG-10 on correction of alternative splicing of MBNL-sensitive exons. For this analysis we selected three alternative exons which showed strong splicing missregulation in *HSA*^LR^, namely *Atp2a1* exon 22, *Titin* exon 5 and *Nfix* exon 7 (Figure 6b). All of them showed significant improvement of the splicing pattern in PO-LNA-CAG-10-treated muscles, and the splicing correction reached almost the level observed in wild-type mice.

Previous studies have shown that misregulated alternative splicing of *Clcn1* results in repetitive action potentials (myotonic discharges) (48). Needle electrode recordings showed suppressive effect of myotonic discharges in PO-LNA-CAG-10-treated TA muscles (Figure 6c). Moreover, similarly to DM1-patient-derived cells, the presence of PO-LNA-CAG-10 did not influence efficient downregulation of CUG^exp^ transcript *in vivo* (Figure 6d). Altogether, our results strongly support the hypothesis that PO-LNA-CAG-10 can invade CUG^exp^ hairpin *in vivo* without induction of strong targeted transcript degradation and efficiently remove MBNLs, and possibly other proteins, from pathogenic sequestration.

## DISCUSSION

Chemical substitutions at the 2′-hydroxyl group with 2′-*O*-Me, 2′-F, 2′-MOE groups or LNA modification often improve the oligomers potency, stability and their overall pharmacokinetic properties (49). The LNA, containing a methylene linkage between the 2′-oxygen and 4′-carbon in the ribose ring, forms a class of modified nucleotides of immense interest. It has been demonstrated that duplex formation by LNA and its target strand is enthalpically favorable because of more efficient stacking of bases in duplexes, resulting in an A-type helical conformation. Biochemical and biophysical studies have shown that LNA has high affinity and specificity for the target strand. Because of these properties, LNA-containing oligomers have been used as probes in DNA sequencing and hybridization techniques (19) but also in RNAi (24) and antisense strategies (21,28,50). In this work, we applied all-LNA oligomers to inhibit RNA/protein interaction. Our results showed for the first time that very short synthetic oligomers composed of only eight or ten LNA subunits (PO-LNA-CAG-8, PO-LNA-CAG-10 and their phosphorothioated derivatives) can localize to cell nuclei, efficiently bind to thermodynamically stable CUG^exp^ hairpin structures both *in vitro* and *in vivo* and inhibit toxic sequestration of nuclear proteins, including MBNLs.

Most of the previously published studies that involved targeting CUG repeats with ASOs have demonstrated that such an approach often triggers degradation of CUG^exp^-containing transcripts, either via nuclear RNase H pathway (12,14) or via an unknown mechanism (15,16), with the exception of morpholino CAG-25 (17). Neither the CAG-25 nor the short LNA-CAGs described in this study caused strong reduction in the level of CUG^exp^-containing transcripts below disease-inducing threshold in cellular and mouse models of DM1 (17). Our results also demonstrate that both types of antisense oligomers have similar potency to reduce the nuclear CUG^exp^ foci and to correct the DM1-specific alternative splicing abnormalities. The fact that all-LNAs are negatively charged and are about three times smaller than CAG-25 suggests that they could be efficiently delivered to affected tissues.

Previous studies showed that clinically promising antisense oligomers are expected to improve the target specificity, cellular stability and immunoresistance compared to RNA-based drugs (19). They mainly include additional modifications such as phosphorothioated linker. In general, the PS-modified oligomers display better affinity, higher nuclease resistance and better pharmacokinetic properties as well as lower toxicity compared to unmodified oligomers (19–23,28). It was reported that the renal clearance and excretion of PS-modified oligomers was significantly lower compared to non-PS oligomers, mainly due to binding plasma proteins by PS-oliogmers with high affinity (51). This binding has also consequences on higher chance of PS-LNA oligomers to enter cells without any carriers and non-specific immune response (45,52). Previously, few strategies using phosphorothioated oligomers were tested in clinical trials (20,22,53). They include phosphorodiamidate-linked 2′*O*Me RNA in exon skipping therapy of Duchenne muscular dystrophy (54) and PS-LNA mixmer against miR-122 for treatment of chronic hepatitis C virus infection (26).

Therefore, to further improve bioavailability of short all-LNA oligomers, we tested the possibility of unassisted delivery of their phosphorothioated derivatives in cell-based models. After long treatment of DM1-cells with high amount, of PS-LNA-CAGs (10 μM), the cellular concentration of the oligomer reached the level sufficient enough to reduce the efficacy of CUG^exp^ foci formation, significantly inhibit MBNLs sequestration and induce alternative splicing correction. These results suggests potential application of these oligomers in systemic delivery in DM1 mouse models. Previously, naked all-PS-LNAs were shown to be efficiently delivered into many tissues by systemic delivery (28,46,47). Future *in vivo* strategies could also utilize oligomer-peptide or oligomer-lipid conjugates in the systemic delivery, as previously described for morpholino CAG-25 (55,56).

Our experiments demonstrated that all-LNAs are stable molecules in cellular environment. In proliferating cells the duration of beneficial effect of PO-LNA-CAG-10 on splicing correction was relatively long and reached ∼20 days in spite of significant dilution of the oligomer after several cell divisions. Moreover, the therapeutic effects in differentiated muscle fibers of *HSA*^LR^ mice lasted minimum 14 days. This is at least twice as long as activity of siRNA directed against CUG^exp^ in the same mouse model (9). Moreover, relatively high 10-μg dose of PO-LNA-CAGs in intramuscular injection did not induce any significant immune-stimulation or muscle degeneration effects, which for such a dose were commonly observed for RNA-based drugs (9) and LNA/DNA gapmers (14) targeting CUG repeats. Our observations indicate that short all-LNAs could be taken into consideration as a promising therapeutic tool for efficient inhibition of pathogenic interaction between RNA and other macromolecules.

## SUPPLEMENTARY DATA

Supplementary Data are available at NAR Online.

SUPPLEMENTARY DATA
