# Supplementary material for: Short antisense-locked nucleic acids (all-LNAs) correct alternative splicing abnormalities in myotonic dystrophy
Source: Nucleic Acids Res. 2015 Mar 9;43(6):3318–31. doi: 10.1093/nar/gkv163 (PMC4381072; doi:10.1093/nar/gkv163)
Supplement: SUPPLEMENTARY DATA [file supp_43_6_3318__index.html]

Short antisense-locked nucleic acids (all-LNAs) correct alternative splicing abnormalities in myotonic dystrophy — Short antisense-locked nucleic acids (all-LNAs) correct alternative splicing abnormalities in myotonic dystrophy — SUPPLEMENTARY DATA 

# Short antisense-locked nucleic acids (all-LNAs) correct alternative splicing abnormalities in myotonic dystrophy

## SUPPLEMENTARY DATA

**Files in this Data Supplement:**

- SUPPLEMENTARY DATA
