# Supplementary material for: Short antisense-locked nucleic acids (all-LNAs) correct alternative splicing abnormalities in myotonic dystrophy
Source: Nucleic Acids Res. 2015 Mar 9;43(6):3318–31. doi: 10.1093/nar/gkv163 (PMC4381072; doi:10.1093/nar/gkv163)
Supplement: SUPPLEMENTARY DATA [file supp_gkv163_nar-02235-y-2014-File008.pdf]

Supplementary Table

Table S1. The list of PCR primers

Transcript name	Sequence 5'-3'	Annealing temperature [°C]	Nr of PCR cycles
APLP2	f:GGAGGAACCAAAGCCTCTCT<br>r:GCTCTCCCACTCCAGATCCT	55	31
KIF13A	f: ACCTGTGCAGCATTCAGGGACAC<br>r: CTCGTCGTTTAATGAGTGCATCTG	55	27
SOS1	f: CAGTACCACAGATGTTTGCAGTG<br>r: TCTGGTCGTCTTCGTGGAGGAA	55	27
PPP3CB	f:AAATTCGAGCAATTGGCAAG<br>r: CAAACCCTTTGCCTCTTCAA	60	29
ECT2	f:TTGGTTCAAGAAGCTGGAAAA<br>r: CAGAATCCTGAAAGTCCGTGA	55	35
PHKA1	f:TGCACACACTTGAGCTTCATGGA<br>r:AAAGTCCACCTCCCCAGACTGGTC	55	33
MBNL1	f:GCTGCCCAATACCAGGTCAAC<br>r:TGGTGGGAGAAATGCTGTATGC	55	23
MBNL2	f:TCCTTTACCAAAGAGACAAGCAC<br>r:CTCAATGCAGATTCTTGGCATTCC	55	25
INSR	f:CCAAAGACAGACTCTCAGAT<br>r:AACATCGCCAAGGGACCTGC	55	30
NCOR2	f:ACACCCACAACCGGAATGAGCCTG<br>r:GGACTTGGCTTTTCGGCTGCTG	55	27
NFIX	f:GAGCCCTGTTGATGACGTGTTCTA<br>r:CTGCACAAACTCCTTCAGTGAGTC	55	25
EIF2AK3	f: GGCTGAAAGATGAAAGCACAG<br>r: TTCCTGAGAATTCCAGTGGTG	55	Real-time
LRP8	f: ACCTGGTGAAGCGGAACTATT<br>r: AGATGGTCTTATTGCCCGAGT	55	Real-time
PAPSS2	f: GAGGTGGCTAAGCTGTTTGC<br>r: TCCACTACCTGGTGGACACA	55	Real-time
CTSA	f: GGCCCTTCAAGATTTCTTCC<br>r: CCCAGAAGGCCATGGTAGTA	55	Real-time
BPGM	f: TGGCTTTGAATCATGGTGAA<br>r: GGAGCAATCCTTTCATTCCA	55	Real-time
DMPK	f:CACTGTCGGACATTCGGGAAGGTGC<br>r: GCTTGCACGTGTGGCTCAAGCAGCTG	55	30
GAPDH	f:CATCAATGGAAATCCCATCAC<br>r:GGTTTTTCTAGACGGCAGGTC	55	23

Supplementary Figures

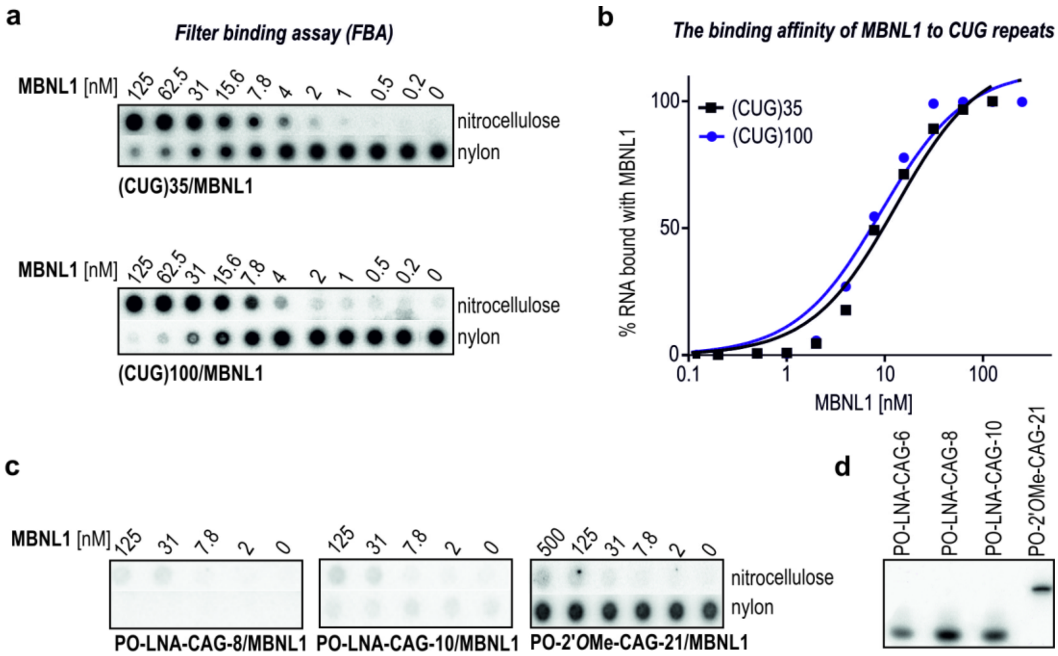

Supplementary Fig. S1. Filter binding assay (FBA) analysis - measuring the interaction of MBNL1 protein with CUG repeat RNAs and CAG repeat oligomers. (**a**) FBA shows similar affinity of MBNL1 to radioactively labeled shorter (CUG)35 and longer (CUG)100 transcripts. Nitrocellulose holds signals derived from the RNA/MBNL1 complexes whereas nylon from free RNA. (b) The quantification of FBA experiment shows affinity of MBNL to both transcripts. (c) FBA was performed to examine the interaction of MBNL1 with short oligomers. PO-LNA-CAG-8 and PO-LNA-CAG-10 are not captured by nylon membrane. Lack of signal on a nitrocellulose membrane indicates that PO-LNA-CAG-8 and PO-LNA-CAG-10 oligomers do not bind to MBNL1 as efficiently as the control. (d) 20% native polyacrylamide gel showing migration of radiolabeled oligomers used in experiments, ~1 nM each.

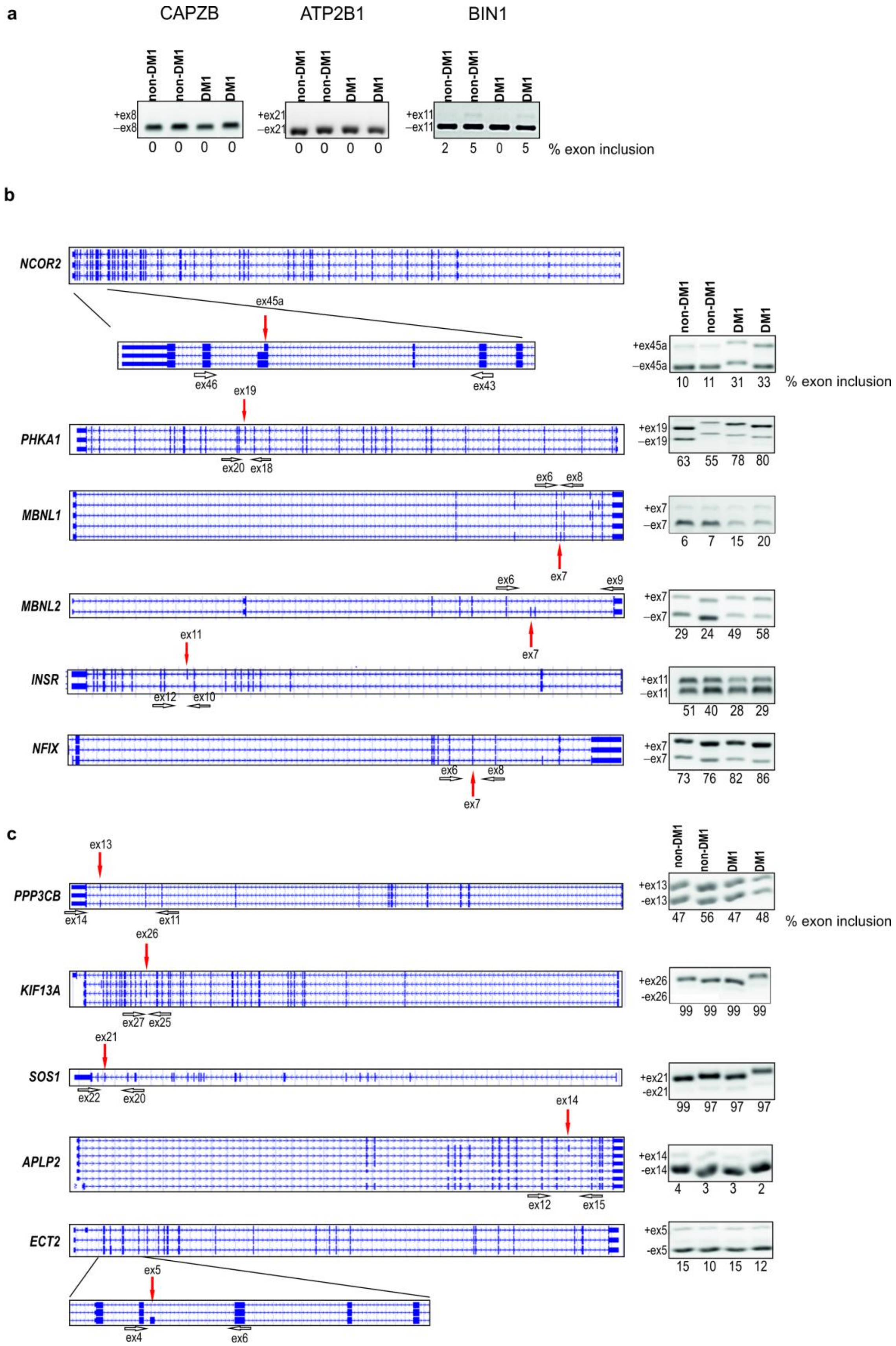

Supplementary Fig. S2. Splicing pattern of selected alternative exons in non-DM1 and DM1 fibroblasts used in this study. (**a**) Examples of RT-PCR assays showing splicing of alternative exons affected in DM1 muscles, which, however, did not show any differences between non-DM1 and DM1

fibroblasts. Two of them, *CAPZB* exon 8 and *ATP2B1* exon 21 were previously shown to be CELF1-dependent. Such exons were excluded from further analyses also due to presence of only one splicing isoform. (**b**) The maps of genes analyzed in this study which have the MBNL-depended alternative exons and (**c**) MBNL-independed exons. Gene diagrams are Refseq sequences from UCSC Genome Browser. Positions of analyzed alternative exons are marked by red arrows. White arrows show localization of RT-PCR primers. The alternative splicing-sensitive RT-PCR assays for two non-DM1 and two DM1 fibroblast cell lines were shown on the right side of each gene map. The percentage of alternative exon inclusion is shown below each lane.

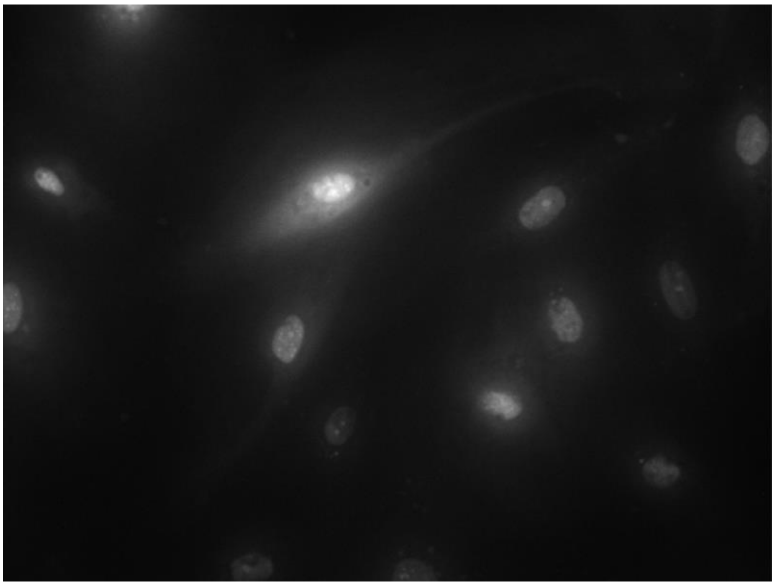

Supplementary Fig. S3. LNA-containing oligomers predominantly localized in cell nuclei. The representative image showing cellular localization of fluorescently labeled (3'-FAM) LNA-containing oligomers after single transfection of fibroblast cells.

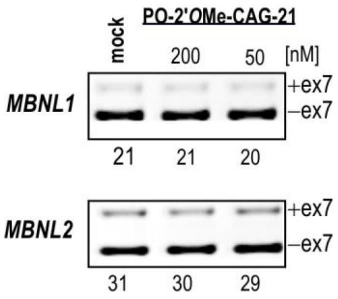

Supplementary Fig. S4. Antisense PO-2'*O*Me-CAG-21 does not improve alternative splicing in DM1 cells. RT-PCR results of alternative splicing changes of *MBNL2* and *MBNL1* transcripts in (CUG)1000 fibroblasts treated with either transfection reagent (mock) or two concentrations of PO-2'OMe-CAG-21 (50 and 200 nM). The percentage of exon 7 inclusion is shown below each lane.

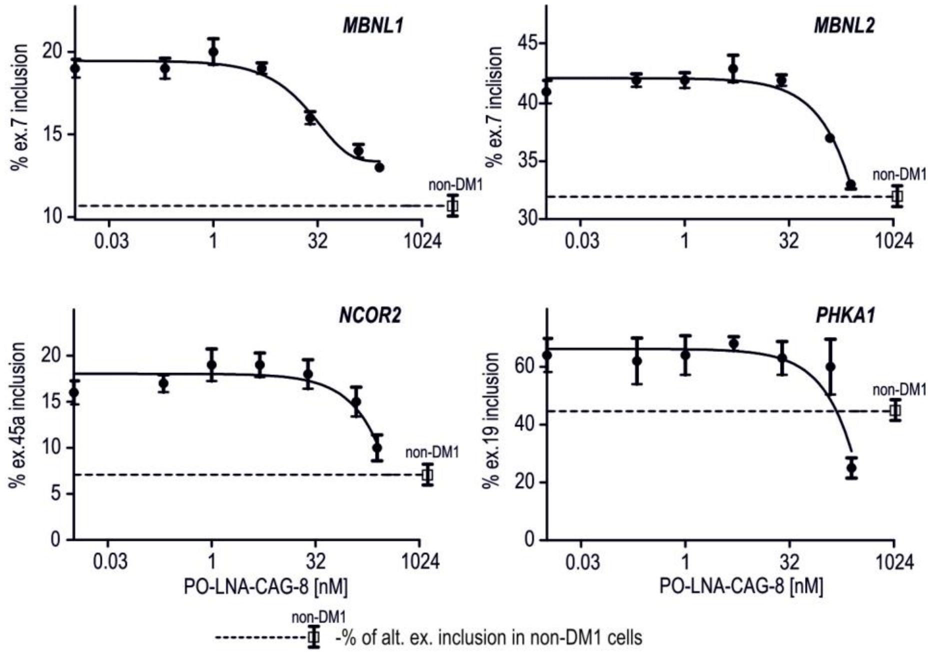

LogEC50	*MBNL1*	*MBNL2*	*NCOR2*	*PHKA1*	*INSR*	*NFIX*
**PO-LNA-CAG-10**	25.0 ± 0.1	5.2 ±0.1	25.1 ±0.2	41.4 ±2.2	4.7 ±0.2	4.9 ±0.2
**PO-LNA-CAG-8**	24.9 ± 0.1	105.4 ±2.4	125.2 ± 0.2	171.3 ±5.6		

Supplementary Fig. S5. PO-LNA-CAG-8 corrects alternative splicing of MBNL1-dependent exons with lower potency. Results of RT-PCR analyses of alternatively spliced exons of *MBNL1*, *MBNL2*, *NCOR2* and *PHKA1* transcripts in (CUG)1000 cells treated with PO-LNA-CAG-8 at concentration range of 0.2-250 nM. The splicing patterns of all tested alternative exons for not treated non-DM1 cells are also shown. The table summarizes the EC_{50} values calculated for each transcript for PO-LNA-CAG-8 and PO-LNA-CAG-10 treated DM1 cells. Other details as described in Fig. 1d.

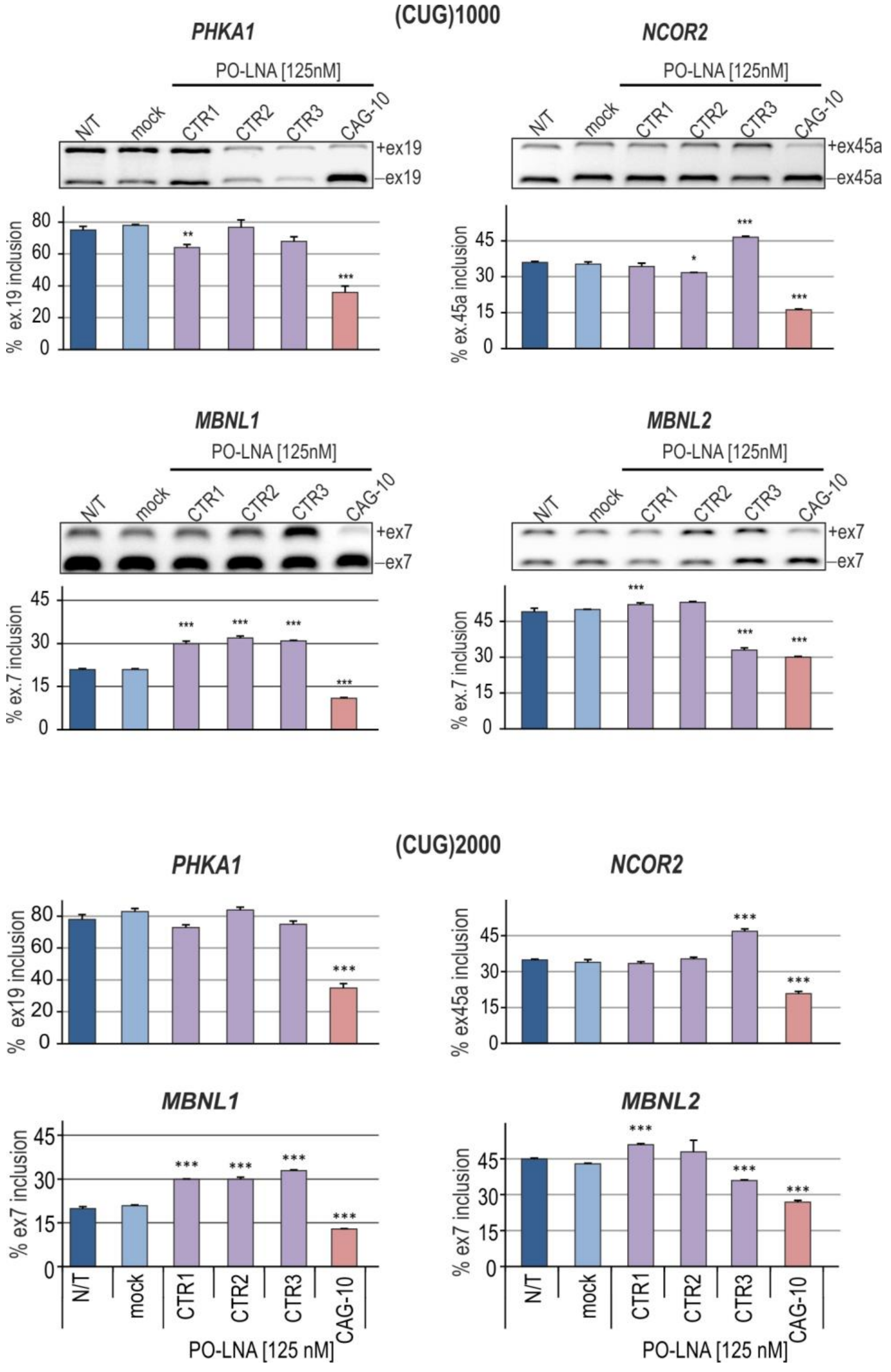

Supplementary Fig. S6. **Control oligomers containing 10 LNA units do not correct alternative splicing of MBNL1-dependent exons.** Results of RT-PCR assays for alternatively spliced exons of *PHKA1*, *NCOR2*, *MBNL1* and *MBNL2* in (CUG)1000 (upper panel) and (CUG)2000 (lower panel) which were not treated (N/T) or transfected with either transfection reagent only (mock) or one of three control PO-LNAs (CTR1, CTR2, CTR3) or PO-LNA-CAG-10 (each at concentration of 125 nM). The results are represented as an average from three independent experiments. *P* values were calculated using two-tailed Student's *t*-test by comparison to results obtained for the same cells treated with transfection reagent only (* $P < 0.05$, ** $P < 0.01$ and *** $P < 0.001$).

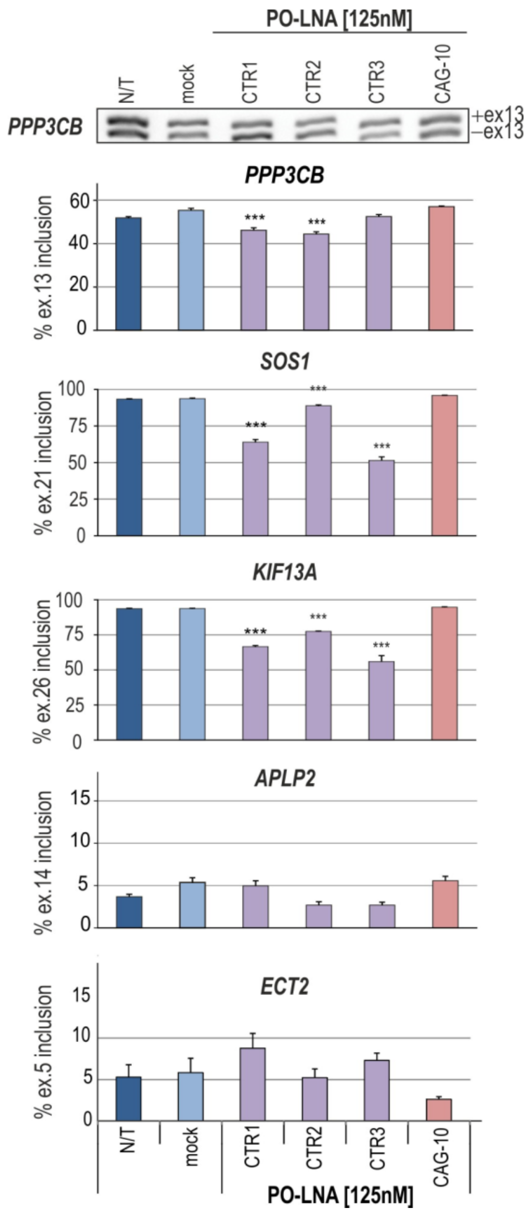

Supplementary Fig. S7. PO-LNA-CAG-10 does not influence the alternative splicing of several exons regulated by other splicing factors. Quantitative analysis of RT-PCR results for alternative exons, whose splicing was previously shown to be regulated by other than MBNL splicing factors. The (CUG)1000 cells were transfected with 125 nM of control PO-LNA oligomers (CTR1, CTR2, CTR3) or PO-LNA-CAG-10. Neither of the analyzed transcripts displayed statistically significant changes of the alternative exon inclusion after treatment with PO-LNA-CAG-10. The results are shown as an average from three independent experiments. P values were calculated using two-tailed Student's *t*-test by comparison to results obtained in cells treated with transfection reagent only (* $P < 0.05$, ** $P < 0.01$ and *** $P < 0.001$).

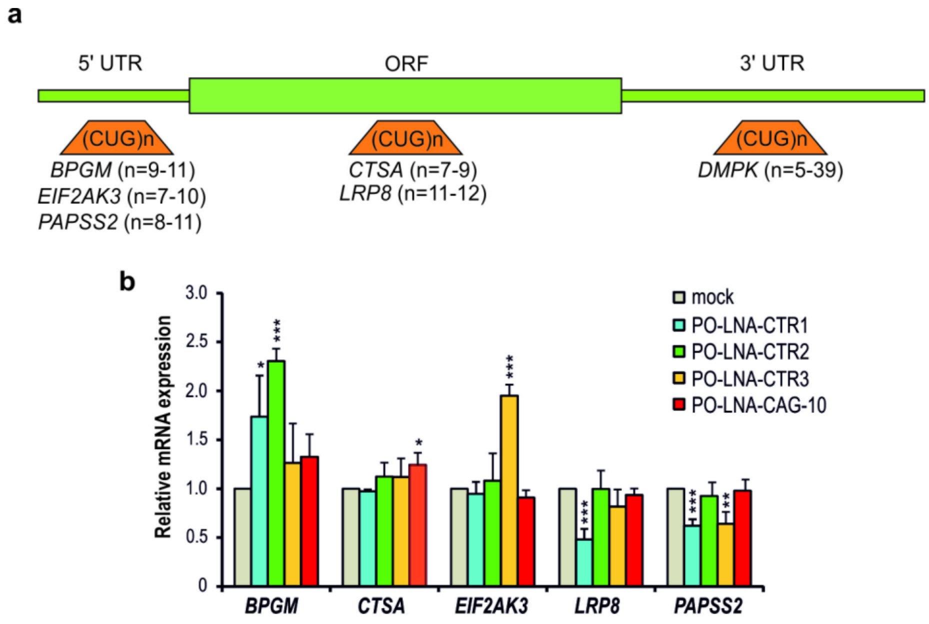

Supplementary Fig. S8. PO-LNA-CAG-10 does not affect the level of transcripts containing short CUG repeats In treated DM1 fibroblasts . (**a**) Localization within mRNA structure and the range of CUG repeat length (from 7 to 12 repeats) in five analyzed transcripts. (**b**) The relative levels of these transcripts in (CUG)1000 cells treated with control LNAs and PO-LNA-CAG-10 were measured by real-time RT-PCR and results were normalized to *GAPDH* mRNA. Results are represented as a fold change in transcript expression, calculated using -ΔΔCt method. Statistical significance was determined using two-tailed Student's *t*-test by comparison of average results from three independent experiments for LNA and mock transfected cells (* $P < 0.05$, ** $P < 0.01$ and *** $P < 0.001$). All results were normalized to mock transfected cells fixed as 1.

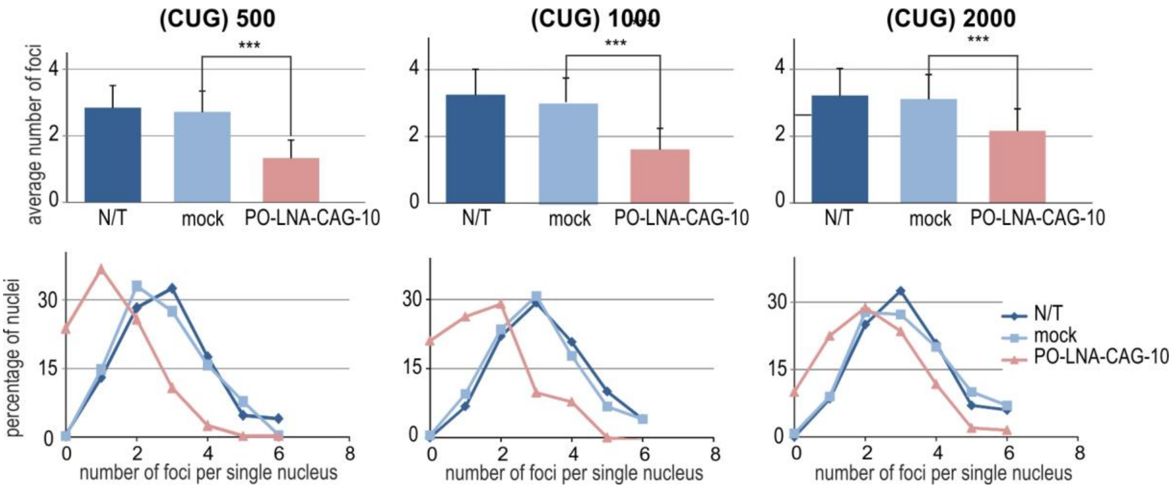

Supplementary Fig. S9. The average number of CUG^{exp} containing foci is significantly decreased in distinct DM1 cell lines treated with PO-LNA-CAG-10. FISH analyses of CUG^{exp} foci in (CUG)500, (CUG)1000 and (CUG)2000 fibroblasts treated with transfection reagent only (mock) or with PO-LNA-CAG-10 (125 nM). The average number of foci per nucleus was calculated for 400 arbitrary selected nuclei.

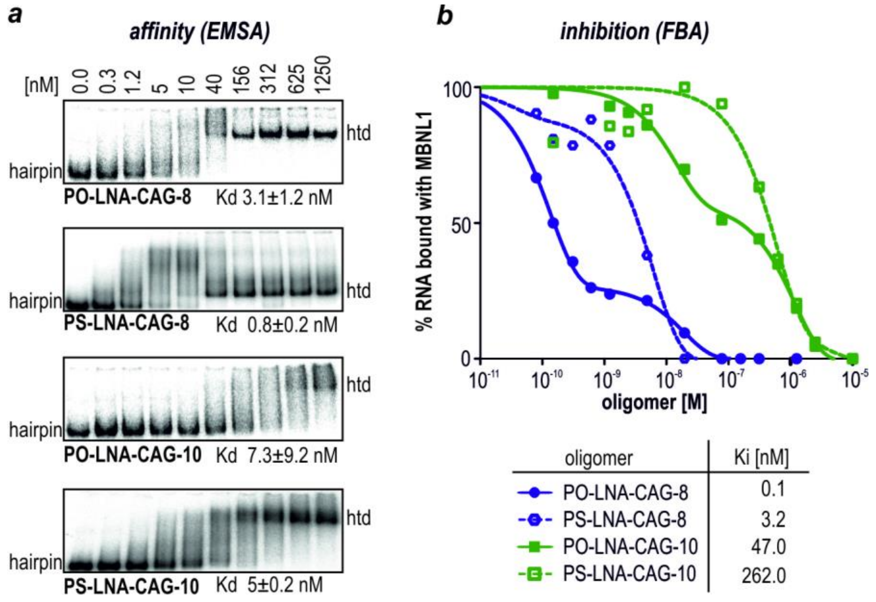

Supplementary Fig. S10. Phosphorothioated LNAs (PS-LNAs) bind to CUG repeat hairpin with the same affinity as unmodified LNAs (PO-LNAs), and efficiently inhibit interactions with MBNL1. (**a**) EMSA experiments for two phosphorothioated and two unmodified PO-LNA-CAGs show only slight differences in the interaction of indicated oligomers with radiolabaled $(CUG)_{100}$ RNA. The K_d value is significantly lower for PS-LNA-CAG-8. (**b**) Unexpectedly, the FBA showed significantly higher values of inhibition constant (K_i) for both PS-LNA-CAGs than for PO-LNA-CAGs.

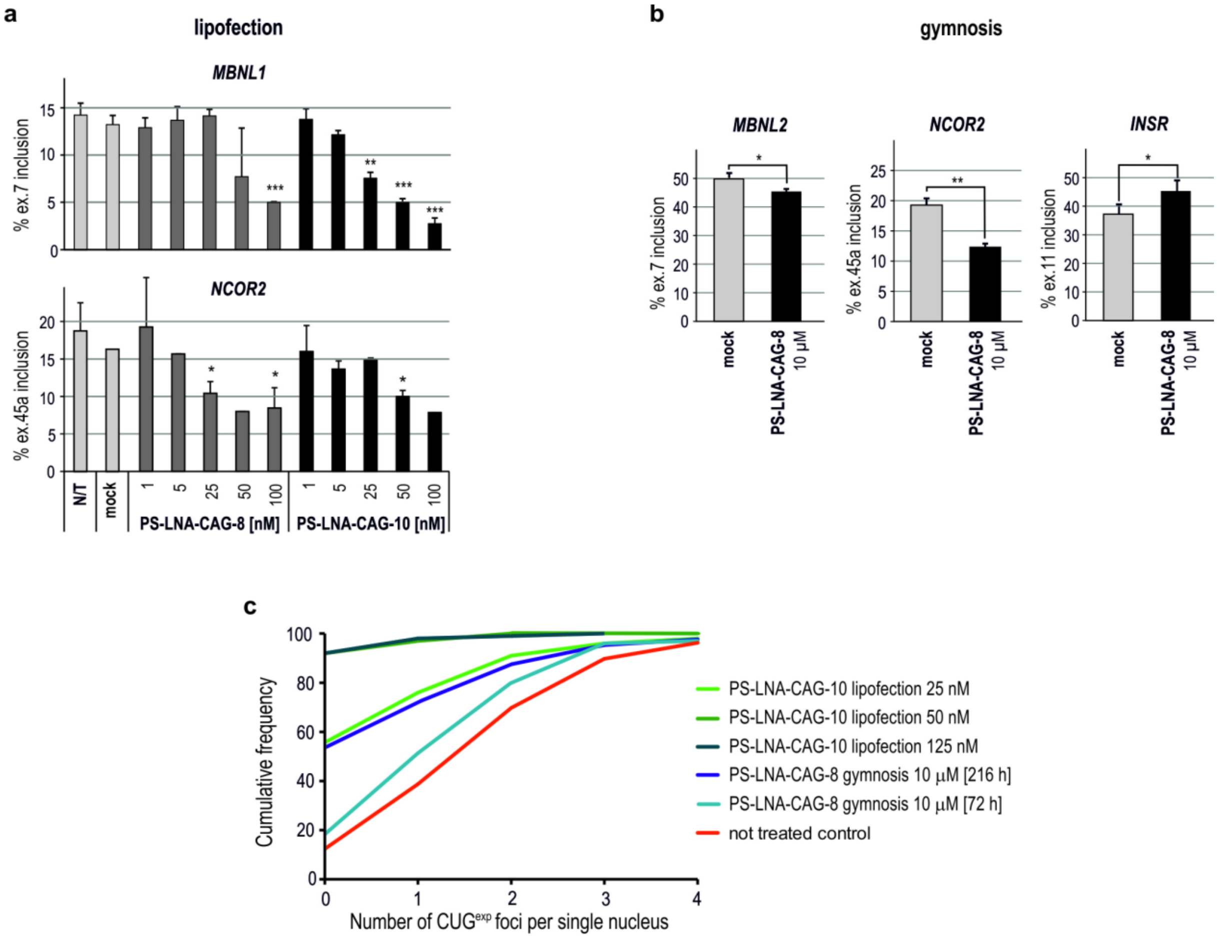

Supplementary Fig. S11. 10 µM of PS-LNAs gymnoticly delivered is as effective as 25 nM PS-LNA delivered by lipofection (**a**) Results of RT-PCR analyses of alternatively spliced exons of *MBNL1* and *NCOR2* in (CUG)1000 fibroblasts transfected with indicated concentrations (1-100 nM) of PS-LNA-CAG-8 or PS-LNA-CAG-10. The results are represented as an average percentage of exon inclusion isoform. (**b**) Results of RT-PCR analyses of alternatively spliced exons of *MBNL2*, *NCOR2* and *INSR* in (CUG)1000 fibroblasts incubated for 216 hours (9 days) in growing medium containing PS-LNA-CAG-8 at the concentration of 10 µM (gymnotic delivery). The results are represented as an average percentage of exon inclusion isoform. (**c**) Distribution of nuclear CUG^{exp} foci based on FISH analysis in (CUG)1000 fibroblasts treated with 25, 50 and 125 nM PS-LNA-CAG-10 delivered by single transfection and 10 µM PS-LNA-CAG-8 delivered gymnoticaly by incubation for 72 or 216 h. The number of foci were determined by counting 100 nuclei (for lipofection experiments) or 300 nuclei (for gymnotic delivery experiments and for untreated cells).
